# Bright Sparks of Single-Atom and Nano-Islands in Catalysis: Breaking Activity-Stability Trade-Off

**DOI:** 10.1007/s40820-025-01978-9

**Published:** 2026-01-12

**Authors:** Xinyu Liu, Suhua Chen, Shenglian Luo, Bo Li, Jiajie Wang, Gaoxia Zhang, Yuqi Zhu, Jianping Zou

**Affiliations:** 1 Key Laboratory of Jiangxi Province for Persistent Pollutants Control and Resource Reuse, School of Environmental and Chemical Engineering, Nanchang Hangkong University, Nanchang, 330063 People’s Republic of China; 2 School of Environment and Energy, South China University of Technology, Guangzhou Higher Education Mega Centre, Guangzhou, 510006 People’s Republic of China; 3 Carbon Neutrality Research Institute of Power China Jiangxi Electric Power Construction Co., Ltd., Nanchang, 330001 People’s Republic of China

**Keywords:** Single-atom catalysts, Nano-islands, Bright sparks, Mechanisms, Interactions

## Abstract

Single-atom catalysts (SACs) are among the most cutting-edge catalysts in the multiphase catalysis track due to their unique geometrical and electronic properties, the highest atom utilization efficiency, and uniform active sites. SACs have been facing an unresolved problem in practical applications: the opposing contradiction of activity-stability. The successful development of single-atom nano-islands (SANIs) cleverly combines the ultra-high atom utilization efficiency of SACs with the confinement effect and structural stability of nano-island structures, realizing the “moving but not aggregation” of SACs, which fundamentally solves this inherent contradiction. Although research on the precise loading of single atoms on nano-islands continues to advance, existing reviews have not yet established a closed-loop cognitive framework encompassing “models-synthesis-high stability mechanisms-high activity essence-applications.” This work fills this critical gap by systematically integrating the basic conceptual models and cutting-edge synthesis strategies of SANIs, focusing on revealing the underlying mechanisms by which SANIs overcome the stability bottleneck of SACs, elucidating the role of nano-islands and their synergistic mechanisms to clarify the high activity essence, and establishing the structure–activity relationship between atomic confinement effects and macroscopic performance, ultimately achieving breakthrough validation across catalytic systems. This review aims to open new perspectives, drive a paradigm shift in understanding the multi-dimensional advantages of SANIs, and thereby spur breakthrough progress in this frontier field.

## Highlights

- Single-atom nano-islands architecture enables “moving but not aggregation” of single atoms, fundamentally overcoming the inherent activity-stability trade-off in single-atom catalysts.
- Systematic synthesis strategies and multi-scale stabilization mechanisms for single-atom nano-islands are detailed, including one-step and two-step approaches, alongside electronic structure modulation via nano-island interactions.
- Single-atom nano-islands demonstrate exceptional performance across diverse catalytic applications, including batteries, clean energy production, chemical synthesis, and environmental catalysis, establishing robust structure-activity relationships.

## Graphical abstract

**Figure:**
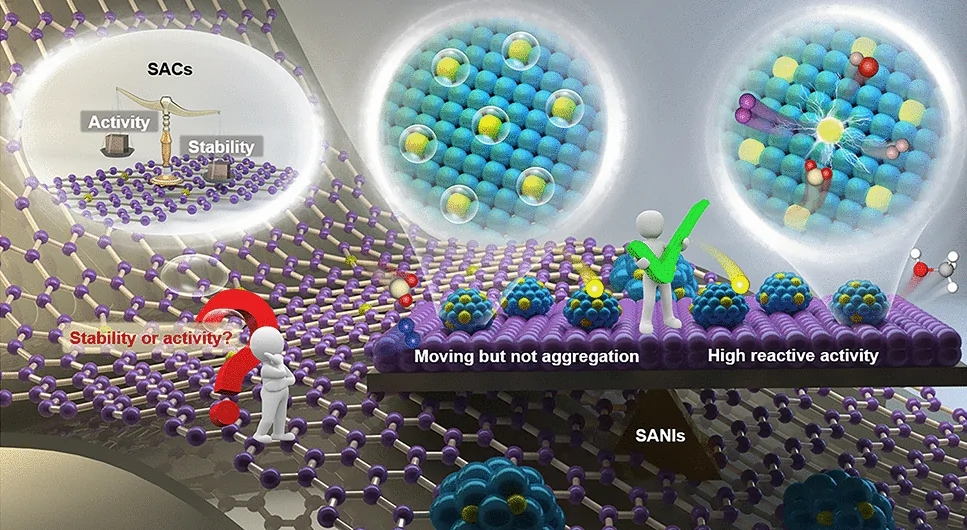

## Introduction

In 2011, Zhang et al. immobilized isolated Pt atoms onto iron oxide (FeO_*x*_) (Pt_1_/FeO_*x*_) to show extraordinary catalytic performance for CO oxidation, and proposed the concept of “single-atom catalysts (SACs)” in the field of heterogeneous catalysis for the first time [1]. As soon as the concept of SACs was proposed, it has rapidly developed into one of the most active research frontiers in the field of multiphase catalysis and attracted extensive attention from both academia and industry [2–10]. The “soul idea” of SACs lies in the individual immobilization of catalytically active metal atoms on support materials through precisely designed ligand/ion interactions between neighboring atoms [11]. This atomic-scale dispersion strategy maximizes the utilization efficiency of each metal atoms, significantly improving both catalytic activity and reaction selectivity while optimizing catalytic process efficiency [12–15].

However, SACs face an inherent thermodynamic dilemma, as their extremely ultra-high surface free energy makes them highly susceptible to atomic agglomeration and sintering under high-temperature or reducing operational conditions, leading to structural collapse and a sharp decline in catalytic performance [16–18]. To address the stability issues of SACs, researchers have explored various strategies, including utilizing surface defects [19–22], using N-doped carbon (CN)/oxide supports [23–28], and employing metalsupport interactions (MSIs)-based electronic/chemical anchoring strategies to stabilize single atoms (SAs) [29–33], to optimize the coordination environment of SAs and enhance their stability. The activity and stability of SACs are inherently contradictory due to their atomically dispersed structure. High activity stems from the unsaturated coordination environment of isolated metal atoms, while stability requires enhanced MISIs. This fundamental contradiction makes it difficult to achieve both high activity and structural robustness in SACs, thereby becoming the core constraint on their development.

This inherent paradox could be fundamentally resolved if SACs were able to achieve “moving but not aggregating” behavior. With this in mind, the researchers designed an innovative nano-island structure that confines active Pt SACs in discrete and defect-rich CeO_*x*_ nano-islands anchored on high surface area SiO_2_ for efficient CO oxidation [34]. This unique structure allowed free migration of Pt SACs within the designated nano-islands and did not cluster across the islands, realizing “moving but not aggregation” of active sites. Remarkably, the Pt atoms maintained atomic dispersion and structural stability even under harsh high-temperature oxidation and reduction conditions. This peculiar structural design enables precise control over the number of metal atoms per nano-island, providing unprecedented flexibility in catalyst design, thereby bypassing the traditional activity-stability trade-off. The core feature of single-atom nano-islands (SANIs), a novel catalytic material system, is the anchoring of isolated metal atoms on the surface of the dispersed nano-island structure or embedded in the nano-islands, resulting in a multistage composite structure of “SACs-nano-island-sea” [35]. Differently, metals in subnanometric metal ensembles-based catalysts exist in subnanoclusters composed of several to a dozen atoms, and atoms are connected by metal bonds to form irregular aggregates [36]. Therefore, the electronic properties of SANIs are primarily regulated by MSIs, and the support can modify their electronic states [37, 38]. Meanwhile, subnanoclusters generate new electronic states due to interatomic interactions. In terms of catalytic performance, SANIs exhibit high atomic utilization efficiency and excellent selectivity, enabling them to fully participate in reactions and perform outstandingly in reactions with high selectivity requirements. This overcomes the limitation of subnanometric metal ensembles-based catalysts, where clusters are prone to aggregation during reactions, leading to a decline in activity.

**Fig. 1:**
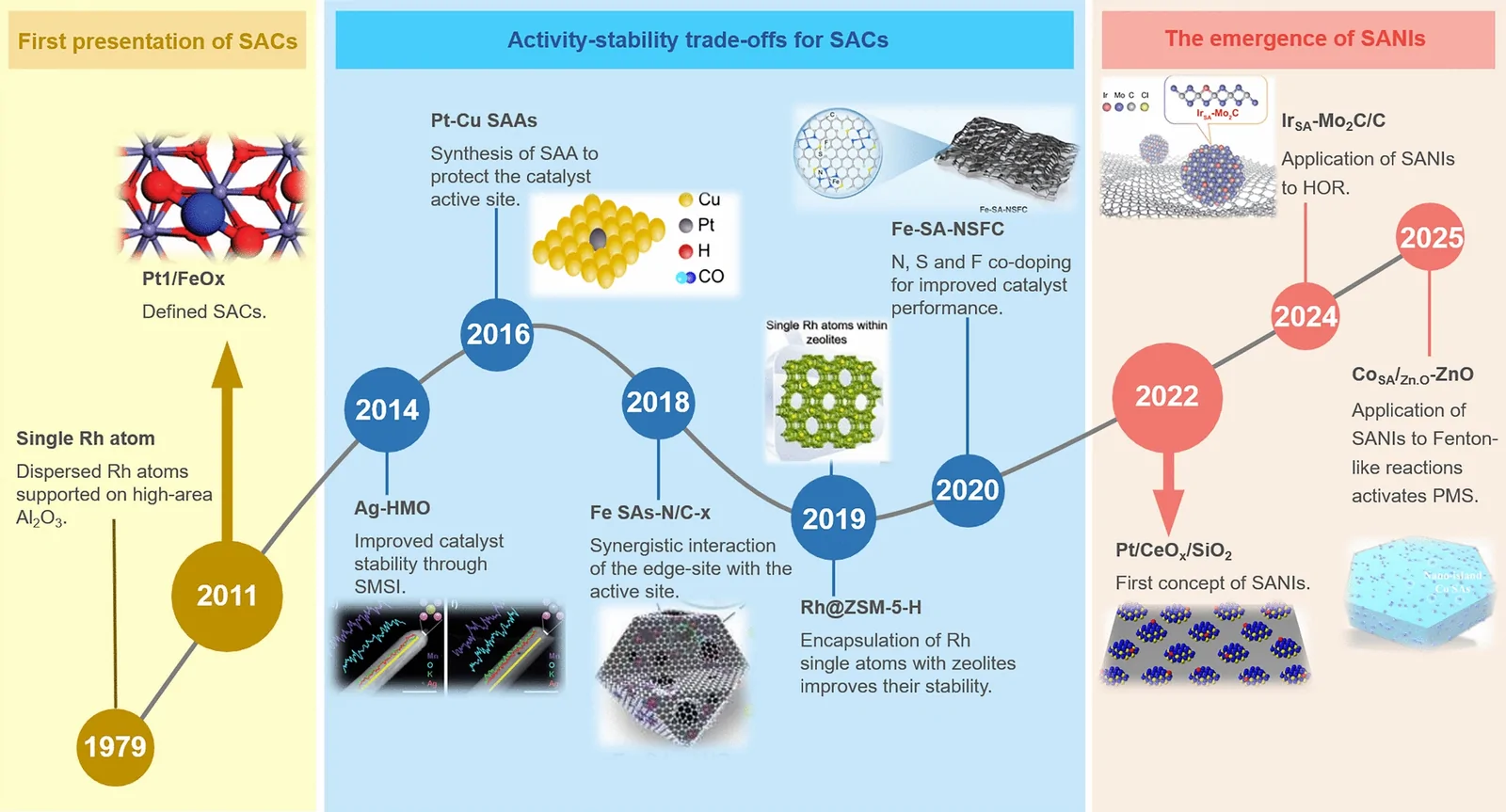
Timeline of the development of SACs to SANIs. Dispersed Rh atoms supported on high-area Al_2_O_3_. Reproduced with permission [43]. Copyright 1979 AIP Publishing. Defined SACs. Reproduced with permission [1]. Copyright 2011 Springer Nature. Improved catalyst stability through SMSI. Reproduced with permission [17]. Copyright 2014 Wiley. Synthesis of SAA to protect the catalyst active site. Reproduced with permission [32]. Copyright 2016 American Chemical Society. Synergistic interaction of the edge-site with the active site. Reproduced with permission [11]. Copyright 2018 American Chemical Society. Encapsulation of Rh SAs with zeolites improves their stability. Reproduced with permission [33]. Copyright 2019 Wiley. N, S and F co-doping for improved catalyst performance. Reproduced with permission [15]. Copyright 2020 Springer Nature. First concept of SANIs. Reproduced with permission [34], Copyright 2022 Springer Nature. Application of SANIs to HOR. Reproduced with permission [44], Copyright 2024 Springer Nature. Application of SANIs to Fenton-like reactions activates PMS. Reproduced with permission [35], Copyright 2025 Springer Nature

The concept of SANIs has since ignited a rapidly expanding research frontier, with an exponential growth of related research in recent years. This paradigm shift demonstrates how spatial constraints and dynamic atomic mobility can synergize to overcome one of the most persistent challenges in catalysis [39, 40]. It also bridges the scientific paradigms of the atomic, nanoscale, and mesoscale levels, which helps bring atomically dispersed metal catalysts closer to practical applications. Previous excellent reviews have already highlighted the potential of SANIs to combine stability and activity, such as Li et al., who explored their scientific significance and application principles in heterogeneous catalysis [41], and Wang et al., who reviewed their unique advantages, design criteria, and latest developments [42]. With the emergence of new research (Fig. 1), the influence of nano-islands on SACs has become increasingly evident, as they determine the synergistic relationship between high stability and high activity through dynamic interface interactions. However, the mechanisms underlying the high stability and exceptional activity of SANIs remain poorly understood. In particular, the mechanisms behind their exceptional stability are precisely what distinguish them from traditional SACs. Meanwhile, the concurrent development of high-precision SAs loading strategies also demands systematic analysis. To establish a closed-loop framework for SANIs spanning model design, precise synthesis, stability mechanisms, activity fundamentals, and application validation, a comprehensive review is urgently needed.

This paper first outlines the basic concepts of SANIs (elemental interactions and material systems) and the three common models within SANIs. Additionally, based on the classification of SACs into precise and chemical assembly within nano-islands, it systematically reviews the most advanced SANIs synthesis strategies, particularly emphasizing the stabilization mechanisms and transformation processes that enable SACs to disperse within nano-islands. Subsequently, the paper focuses on the stability mechanisms of SANIs, systematically revealing the intrinsic mechanisms of that SANIs models overcome the SACs stability bottleneck in different application scenarios. It then delves into the role of nano-islands and their synergistic mechanisms to elucidate the fundamental reasons behind the high activity of SANIs. Finally, the paper discusses the representative advancements achieved by SANIs in the field of catalysis from the perspective of various catalytic reaction mechanisms. Finally, the paper presents concise conclusions and explores the prospects and challenges of SANIs in the field of catalysis. This review aims to open up new perspectives and promote a paradigm shift in the understanding of the multi-dimensional advantages of SANIs, thereby driving breakthrough progress in this frontier field.

## State-of-the-Art of Generating SANIs

The central challenge in the study of SACs is how to stabilize SACs while maintaining catalytic activity. SANIs utilize nano-islands structures to maintain the mobility of SACs while preventing their aggregation, thereby achieving atomic-level stable dispersion. The interactions between nano-islands and their supporting matrix (the “sea” component) have fundamental similarities across systems, thus this study prioritized the precise assembly and chemical assembly of SACs within nano-islands. The current synthesis routes for the assembly of nano-islands and SACs in the presence of the support mainly involve one-step or two-step approaches. This section begins with an overview of the basic concepts and compositions of SANIs, including common material compositions (e.g., metal oxides and carbides) and their structural advantages (e.g., confinement effects and defect-rich surfaces) (Table 1). It then focuses on the differentiated advantages of SANIs compared to other cutting-edge SACs systems, highlighting their dynamic stability and synergistic catalytic properties. Building on this foundation, the theoretical framework of existing SANIs models is analyzed to deepen the understanding of the SANIs concept. The state-of-the-art synthesis strategies for SANIs are then described in detail, with special emphasis on the stabilization mechanisms and conversion processes that enable SACs to be dispersed in nano-islands.

**Table 1:** Summary of SANIs material composition and its structural advantages

Catalysts	Nano-island size and supports SSA	Nano-island characteristics	Supports characteristics	Refs
Ru/WC_*x*_/Amorphous carbon	2–5 nm 154.2 m^2^ g^−1^	1. Microspheres morphology formed from layered nano­sheets; 2. Good adsorption ability	A robust high surface area amorphous carbon support	[45]
Ru_1+n_/Co/MMO	1.17 nm /	Confined Co NIs: improving the affinity of the active sites to provide a dominant adsorption site for key intermediates	MMO (Mixed metal oxide): 1. Diversity of crystal structures; 2. High SSA	[46]
Pt_1_/SnO_2_/UiO-66-NH_2_	1–2 nm /	1. Provides high stability and strong interaction; 2. The Sn^2+^ can induce self-oxidation to form SnO_2_, thereby reducing noble metal ions in situ	MOFs: 1. High SSA; 2. Offers rich pore space and tailored microenvironment	[47]
Ru SA/SnO_2_/C	average 2 nm /	Excellent adsorption capability of OH_ad_	/	[48]
Pt_SA_/MoS_2_/rGO	/	1. Two-dimensional construction; 2. High stability; 3. Platinum-like activity; 4. Controllability	1. High SSA; 2. Highly conductive	[49]
Pt/CeO_*x*_/DMS	/ 451 m^2^ g^−1^	1. Oxygen vacancies and lattice distortion; 2. Structural stability	1. Large SSA; 2. Large pore volume	[50]
Pt_SA_/Mo_2_C/NC	about 2 nm /	Noble metals like d-band contraction electronic characteristic of Mo_2_C	1. High SSA; 2. Open mesopores	[51]
Ir_SA_/Mo_2_C/C	average 2.4 nm /	1. Pt-like electronic structure; 2. High stability and strong interaction with PGMs	/	[44]
Pt_1_Mo_1_/Ni_3_S_2_/NF	less than 5 nm /	High stability	Large SSA	[52]
Pt_1_/FeO_*x*_/CN	average 1.07 nm 863 m^2^ g^−1^	1. The surface has a lot of defect sites. 2. Strong interaction with Pt	1. Large SSA; 2. Abundant defective sites; 3. Highly conductive	[53]
PtCl_2_/Au(111)/GDY	2.37 nm /	Appropriate d-band states and the stabilizing effect of Au(111)	1. Uniformly distributed microporous and tunable electronic characteristics; 2. Special charge distribution inhomogeneity and rich conjugated alkyne characteristics	[54]
Pt/CeO_*x*_/SiO_2_	less than 2 nm 278 m^2^ g^−1^	1. Excellent redox and oxygen storage properties; 2. Affinity for metal atoms exceeds that of supports	1. High SSA; 2. Structural stability; 3. Commercial availability	[34]
Pt_1_/FeO_*x*_/SBA-15	less than 5 nm /	Strong interactions with Pt	1. High thermal stability; 2. Large SAA	[55]
Pt/InCeO_*x*_/SiO_2_	about 2 nm 743 m^2^ g^−1^	1. Strong interactions with Pt; 2. Inhibition of In^0^ production	1. Large SSA; 2. High thermal stability; 3. Mesoporous structure	[56]
Ir_1_/P_*x*_/In_2_O_3_	about 10 nm /	Exceptional H_2_ dissociation ability	High stability	[57]
Co_SA/Zn.O_/ZnO	/	1. Structural stability; 2. Strong synergistic effects with Co SACs	1. High SSA; 2. pH control	[35]
Pt_1_/POMs/PC	average 0.75 ~ 0.8 nm	1. Provides multiple hydrogen transfer sites; 2. Strong coordination capability; 3. Serves as a channel for hydrogen spillage	1. Structural stability; 2. Highly conductive; 3. Sub-nanopore confinement of POMs and Pt	[58]
Pt_SA_/CeO_2-*x*_/rGO	/	1. Oxygen vacancies; 2. High stability	1. High SSA; 2. Highly conductive; 3. High stability	[59]
Pt-Cl/CeO_x_/SiO_2_	/	High structure stability	High-area porous	[60]
Pt_1_/CeO_x_/SiO_2_	~ 2 nm /	1. Crystalline; 2. Showed a higher concentration of Ce^3+^ andmore oxygen vacancies	1. High SSA; 2. Structural stability	[61]
Pt/CeO_x_/SiO_2_	About 3 nm /	1. Limit the Pt atoms; 2. High stability	High-area porous	[62]

### Concepts and Advantages of SANIs

#### Concepts and Components of SANIs

SANIs are a class of materials that achieve atomic-level dispersion of active sites through a nano-islands-sea (supports) binary structure, thereby forming a three-level composite structure of “SACs-nano-islands-sea.” Their core structural characteristics comprise three inseparable elements: active metal atoms, nano-islands, and supports. This design cleverly combines the high atom utilization of SACs with the domain-limiting effect and stability of nano-island structures, which creates great opportunities for catalytic technology and makes atomically dispersed metal catalysts a big step forward to practical applications.

In SANIs, the “sea.” as the support of the entire catalyst, not only as a physical support for the structure, but also as a key component for achieving strong anchoring of SACs and nano-islands as well as catalyst stability. For the whole structure, the secondary support plays the role of stabilizing the “sea” and ensures the uniform dispersion of the nano-islands, thus improving the apparent activity. The “sea” generally possesses the following properties in the selection of materials: (i) non-metallic oxides. Providing large specific surface area (SSA) and abundant non-metallic stabilization, they can be used to disperse and stabilize the nano-islands for better SACs dispersion; (ii) carbon-based materials. Provide high electrical conductivity and facilitate efficient electron transport; and (iii) metal oxides. Amphiphilic materials that can maintain a reaction-specific pH environment for enhanced stability.

Nano-islands are important bridges between atoms (SACs) and mesoscopic scale (sea), and they are mostly dispersed stably on the surface of supports or embedded in supports in the form of isolated islands, which induces SACs to anchor in their cavities, realizing the design concept of “moving but not aggregation”. The design of nano-islands consists of two key dimensions: (i) at the atomic level, nano-islands need to provide more dangling bond structures/SACs cavities/induced electrostatic adsorption with different charges from SACs to realize the precise localization of SACs on nano-islands; and (ii) at the mesoscopic level, uniform dispersion over the sea to optimize mass transfer and stability. Materials selection must comprehensively consider anchoring capability (e.g., defect density), electronic transport properties (e.g., conductivity), and environmental tolerance (e.g., corrosion resistance). Based on this, metal oxides have become the mainstream nano-islands materials. According to existing research statistics (Table 1), nano-island sizes typically range from 1 to 5 nm in lateral/longitudinal dimensions, with special three-dimensional (3D) structures extending up to 10 nm. Their morphological features primarily include three basic configurations: planar (two-dimensional (2D) extension), 3D (spherical/cubic), and core–shell (functional core@porous shell). Based on the degree of interface contact, they can be categorized into surface-adhered, partially embedded, and fully embedded types. These three configurations collectively form the diverse design framework of nano-islands.

#### Advantages of SANIs

SACs generally face challenges such as difficulty in balancing activity and stability, agglomeration at high loadings, limited control over electronic structure, and susceptibility to poisoning of active sites. Currently, there are also many different types of promising SACs that can address these issues, but each has its own advantages and disadvantages. Carbon-based substrates, though highly active and conductive, struggle to suppress high-loading agglomeration due to insufficient chemical stability [63]. MOF-based substrates can achieve high loading and coordination control, but they are prone to collapse under harsh conditions, making their preparation relatively complex [64]. Metal oxide-based substrates suppress agglomeration and induce defects through strong MSIs, but conductivity defects and the risk of phase transitions under extreme conditions limit their application [65]. Single-atom alloys (SAAs) combine selectivity and stability, but their SAs ratio requires precise control, and high loading easily forms metal clusters [66, 67]. In contrast, SANIs achieve multi-dimensional breakthroughs through their “SAs-nano-islands-support” hierarchical structure: (i) they pioneer a “moving but not aggregation” mode through a synergistic mechanism of physical confinement and defect anchoring, completely suppressing agglomeration while maintaining high activity of unsaturated coordination; (ii) they precisely regulate the electronic structure of SAs via nano-islands to optimize the adsorption energy of intermediates for diverse reactions; (iii) by designing selective adsorption sites to inhibit toxin occupation and block carbon buildup; (iv) through innovative serial active center design to reconfigure complex reaction pathways; and (v) by flexibly adopting one-step or two-step methods to simultaneously achieve nano-island size control and high-loading dispersion of SAs. Therefore, SANIs establish significant advantages in terms of activity-stability balance, reaction universality, and overall performance.

### Different Structural Models of SANIs

**Fig. 2:**
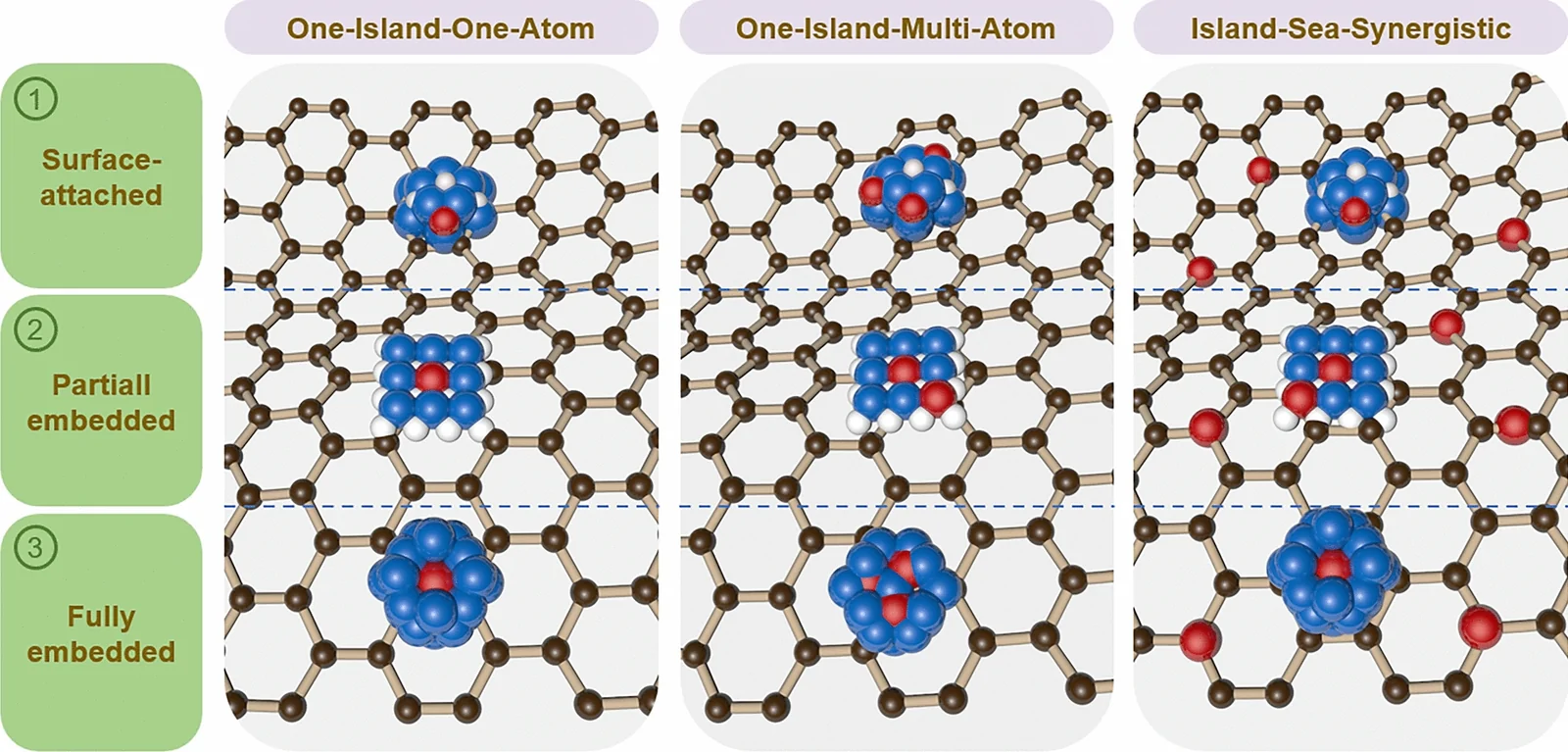
Three typical types of SANIs

SANIs, as a novel atomic-nanocomposite catalytic system, achieve dynamic confinement and functional enhancement of SAs through their unique nano-islands structure, offering an innovative approach to resolving the trade-off between activity and stability in traditional SACs. Based on atomic distribution patterns and structural characteristics [41], SANIs can be categorized into three typical models: “one-island-one-atom.” “one-island-multi-atom.” and “island-sea synergistic” (Fig. 2). Each structure possesses unique scientific significance and application potential.

#### One-Island-One-Atom

In the “one-island-one-atom” architecture, a single metal atom is precisely anchored to an isolated nano-island, forming highly dispersed active sites, its core “moving but not aggregation” property combines dynamic catalytic advantages with high stability. A single metal atom can freely migrate within its nano-island, which may induce further optimization of the coordination structure and electronic configuration of SACs, serving as the source of its high activity. Taking defect-rich CeO_*x*_ nano-islands as an example, the diverse vacancy sites in defect-state nano-islands provided dynamic optimization pathways for Pt atoms, enabling them to locate at the most favorable coordination sites, thereby significantly enhancing catalytic activity [34]. Meanwhile, strong Pt-O bonds anchored Pt atoms on CeO_*x*_, enabling precise control over the stability of Pt metal atoms on CeO_*x*_ nano-islands. The physical isolation effect of isolated nano-islands effectively restricted inter-island atomic migration, completely blocking cluster aggregation pathways. In fact, except to the interactions between nano-islands and SAs, the interactions between nano-islands and the supports also contribute to the stability of the “one-island-one-atom” configuration. Thermodynamically driven electron redistribution achieves equilibrium between metal atoms and supports, ensuring uniform dispersion of the nano-islands [68]. Notably, when the size of the nano-islands is sufficiently small and the density is sufficiently high, the distribution of SAs tends to densify, ultimately achieving a synergistic leap in catalytic performance [41].

#### One-Island-Multi-Atom

The “one-island-multi-atom” model significantly enhances the density of active sites and overall catalytic activity by densely arranging multiple metal SAs on a single nano-island. Compared to traditional high-density SACs, its core advantage lies in the localized effect of the nano-islands, which induces electronic synergistic effects between a specific number of metal atoms. A strategic design schemed for Pt SAs and Ir SAs on nano-islands at an appropriate density have confirmed its feasibility [44, 53, 59]. It is worth noting that excessively high densities can lead to the aggregation of SAs into atomic clusters [69, 70]. While the formation of some metal bonds (e.g., M-M) can optimize electronic structure and enhance specific reaction selectivity, excessive aggregation reduces atomic utilization [71]. In this case, the physical confinement effect of the nano-islands effectively suppresses further aggregation into larger-sized nanoparticles, thereby achieving synergistic enhancement between atomic clusters and nano-islands [62, 72–74]. This mechanism, which balances controlled aggregation with dynamic confinement, endows the architecture with high activity and stability, making it an ideal platform for complex catalytic systems such as multi-step organic synthesis. Therefore, SANIs can be extended to utilize the synergistic effects of atomic clusters and nano-islands, which may bring further innovations to this field.

#### Island-Sea-Synergistic

The core advantage of the “island-sea synergistic” structure lies in its integration of synergistic catalytic mechanisms between different active sites. As an important model, this structure achieves efficient bifunctional catalysis by loading metal atoms with differentiated coordination environments onto the supports (sea) and nano-islands, respectively. The different coordination environments of active metal atoms on the support and nano-islands can form efficient synergistic effects [75]. For example, Yang et al. developed a Pt SAs and PtCo alloy systems that significantly enhanced catalytic performance through synergistic interactions between the two sites [76]. In this architecture, the PtCo nano-island and Co SAs support (sea) system achieved optimized water splitting through proton and hydroxide overflow synergistic interactions between the nano-island (oxygen evolution reaction (OER) active site) and the sea (hydrogen evaluation reduction (HER) active site). Notably, recent breakthrough research has delved deeper into the synergistic essence of the “island-sea synergistic” structure, specifically the synergistic interaction between the sea and island components. This catalyst leveraged the “island-sea synergistic.” using small ZnO nano-islands to confine and stabilize Co SAs, while the vast ZnO substrate (sea) maintained a neutral microenvironment for the reaction system, enabling stable catalytic activity [35]. This multi-active center integration strategy not only maximizes atomic utilization but also precisely regulates multi-step reaction pathways, simultaneously achieving high reaction rates and selectivity in complex electro-oxidation reactions, opening new dimensions for multi-phase catalytic design.

### Latest SANIs Synthesis Strategies

#### One-step Synthesis

The one-step synthesis strategy has emerged as a simplified approach for fabricating SANIs, typically following a “Stage I: precursor loading/support (sea) → Stage II: precursor transformation” pathway. Due to significant differences in chemical properties and functional roles between nano-islands precursors and SACs precursors, their interaction mechanisms also vary greatly. This inherent differentiation has prompted extensive research focusing on three critical aspects: (i) precise loading strategies for dual-component precursors; (ii) interfacial interactions among precursors and between precursors-support; and (iii) phase evolution dynamics during subsequent transformation processes. Accordingly, this section systematically examines prevalent methodologies employed in both stages (I and II) and evaluates their impacts on the resultant physicochemical characteristics of SANIs, including atomic dispersion efficiency, island morphology control, and catalytic interface optimization.

**Fig. 3:**
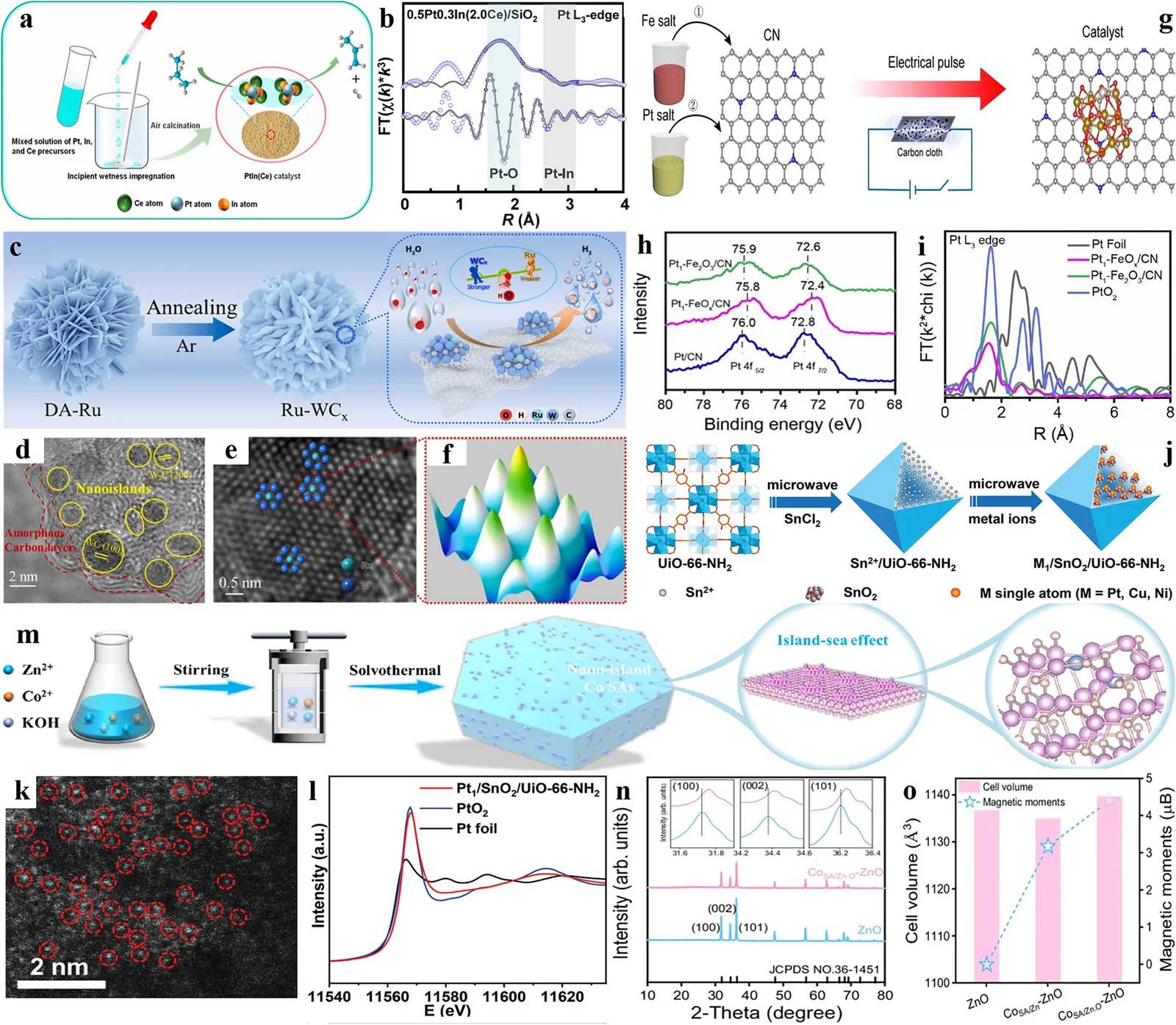
One-step synthesis of SANIs and their characterization techniques. **a** Demonstration of the Synthesis Process of PtIn(Ce) Cluster Catalysts. **b** Pt L_3_-edge EXAFS (points) and curve fit (line) of the 0.5Pt0.3In(2.0Ce)/SiO_2_ catalysts. Reproduced with permission [56], Copyright 2024 American Chemical Society. **c** Synthesis pathway of Ru/WC_*x*_. **de** HRTEM images over Ru/WC_*x*_. **f** 3D-scanning intensity distribution from the region marked with a red rectangle in Fig. 3e. Reproduced with permission [45], Copyright 2024 Elsevier. **g** Synthesis pathway of Pt_1_-FeO_*x*_/CN. **h** Pt 4*f* XPS of the samples. **i** R-space EXAFS of Pt L_3_ edge. Reproduced with permission [53], Copyright 2022 Wiley. **j** The process of microwave-assisted synthesis of M_1_/SnO_2_/MOF (UIO-66 as a representative). **k** Aberration-corrected HAADF-STEM corrected for aberrations show Pt_1_/SnO_2_/UiO-66-NH_2_, with Pt SAs marked through red dashed circles. **l** The Pt L_3_-edge XANES spectra for different samples. Reproduced with permission [47], Copyright 2022 Wiley. **m** Synthesis procedure of Co_SA/Zn.O_ZnO. **n** XRD image over ZnO and Co_SA/Zn.O_ZnO (inset: magnified view showing (100), (002), and (101) crystal planes). **o** Comparison of cell volume and magnetic moment across the three configurations. Reproduced with permission [35], Copyright 2025 Springer Nature

The impregnation-pyrolysis method exemplifies a robust approach for fabricating SACs within the “precursor loading → structural transformation” framework. A seminal study by Wang et al. [56] demonstrated this by dispersing Pt SACs in islands through the impregnation of SiO_2_ support in mixed metal salts (Pt, Ce, In), followed by pyrolysis (500 °C) of the dried precursor to yield 0.5Pt*ₓ*In(_*y*_Ce)/SiO_2_ catalysts (Fig. 3a). During the synthesis of the catalyst, the authors cleverly utilized the constraints between Ce and In. In^3+^ in the _*x*_In*(*_*y*_Ce) nano-islands could assist in the dispersion of Pt atoms, but it was highly susceptible to reduction to In^0+^ to produce PtIn alloys. Interestingly, the introduction of Ce could weaken the alloying ability between Pt and In (Fig. 3b), promoting the formation of low-valent isolated Pt^δ+^ sites (in the presence of Pt-O bonding) while generating ultrasmall InCeO_*x*_ nano-islands. The structural robustness of the Pt^δ+^ sites was achieved through strong electronic interactions between Pt^δ+^ and InCeO_*x*_ nano-islands. To prepare highly dispersed and stable SACs, Chen et al. strategically employed tungsten carbide (WC_*x*_) as the nano-islands and converted the dopamine (DA)-Ru precursor into Ru/WC_*x*_ composites by a calcination (900 °C) process (Fig. 3c) [45]. High-resolution transmission electron microscopy (HRTEM) images (Fig. 3d, e) showed that the Ru/WC_*x*_ nano-islands were distributed on an amorphous carbon “micro-sea.” a continuous graphitized carbon shell that accelerated charge transport and improved the stability of Ru sites. This hierarchical structure not only facilitated efficient charge transport, but also enhanced the structural stability of the active sites. Most importantly, the Ru SACs were confined within the WC_*x*_ lattice, which effectively inhibited their cross-islands migration (Fig. 3f), and the 3D scanning intensity distribution well formalized the dispersion of the Ru SACs.

The electrical pulse method has emerged as a transformative strategy for the synthesis of SACs, utilizing its unique capabilities in low-temperature processing, dynamic coordination modulation, and energy-efficient operation [77]. Building on these advantages, the impregnation-electrical pulse method is now being extended to fabricate SANIs with atomic-level precision. A representative example involved the use of Zif-8-derived NCN) as -doped CN supports and the sequential impregnation of Fe and Pt salts onto the CN surface, after which Pt_1_-FeO_*x*_/CN catalysts were prepared applying an ultra-large current pulse for a duration of ≈ 1 s (Fig. 3g) [53]. Pt and Fe atoms were overlapped within FeO_*x*_/CN, confirming that the electrical pulse process did not affect their segregation. The Pt binding energies in Pt₁-FeO*ₓ*/CN was reduced by about 0.2 eV compared to that of Pt/CN (Fig. 3h), suggesting that the interaction of the Pt SACs with FeO*ₓ* resulted in a reduction of the Pt valence (Fig. 3i). The electrical pulse technique has shown remarkable versatility in the successful synthesis of Pt SACs dispersed on different oxide clusters (MnO_*x*_, CoO_*x*_, and SnO_*x*_). A question worth pondering is whether the order in which the nano-islands and SACs precursors are added affects the dispersion and stability of the SACs? Or is there some kind of involvement between the two during the conversion? In order to clear the layers, Sui et al. explored the effect of Sn^2^⁺ pre-loading on the formation of Pt SACs by utilizing impregnation-microwave-assisted preparation of M_1_/SnO_2_/UiO-66 (M = Pt, Cu, and Ni) (Fig. 3j) [47]. Pre-loading Sn^2+^ coordinated with Zr-oxo clusters via Sn–OZr bonds to form well-dispersed Sn^2+^ (Fig. 3k), facilitating the subsequent redox/hydrolysis reaction with the Pt precursor. This stepwise process enabled Pt SACs deposition as stable on SnO_2_ within UiO-66 cavity, evidenced by X-ray absorption near edge structure (XANES) spectra showing highly oxidized Pt states (Fig. 3l). In contrast, direct mixing of UiO-66 with Pt or Pt and Sn precursors under the same conditions produced Pt NPs rather than SACs, highlighting the important role of sequential pre-loading of Sn^2+^ to achieve atomic dispersion.

Along with the ongoing development of SANIs, recent studies have successfully prepared ZnO and ZnO nano-islands encapsulating Co SACs (Co_SA/Zn.O_ZnO) using the simplified method of “one-step hydrothermal” (Fig. 3m) [35]. X-ray diffraction (XRD) analysis confirmed the excellent crystallinity of ZnO, while clearly demonstrating the atomic-level dispersion of Co SAs within nano-islands (Fig. 3n). Notably, unlike conventional interstitial doping modes, Co SAs achieved stable incorporation by replacing Zn and O atoms in the ZnO lattice. Such unique substitution mechanism induced the largest cell volume (Fig. 3o), creating an optimized electronic environment.

#### Two-step Synthesis

While one-step synthesis offers simplicity in preparing SANIs, it faces limitations in controlling metalprecursor interactions and achieving precise SACs loading. This drives the development of two-step strategies following a “Stage I: nano-islands/support → Stage II: precursor loading → Stage III: precursor transformation” sequence. Crucially, the precursor-loading methodology in Stage II dictates SACs positioning accuracy. This section will systematically evaluate the Phase I synthesis methodology and its significant impact on the physical and chemical properties of customized SANIs.

**Fig. 4:**
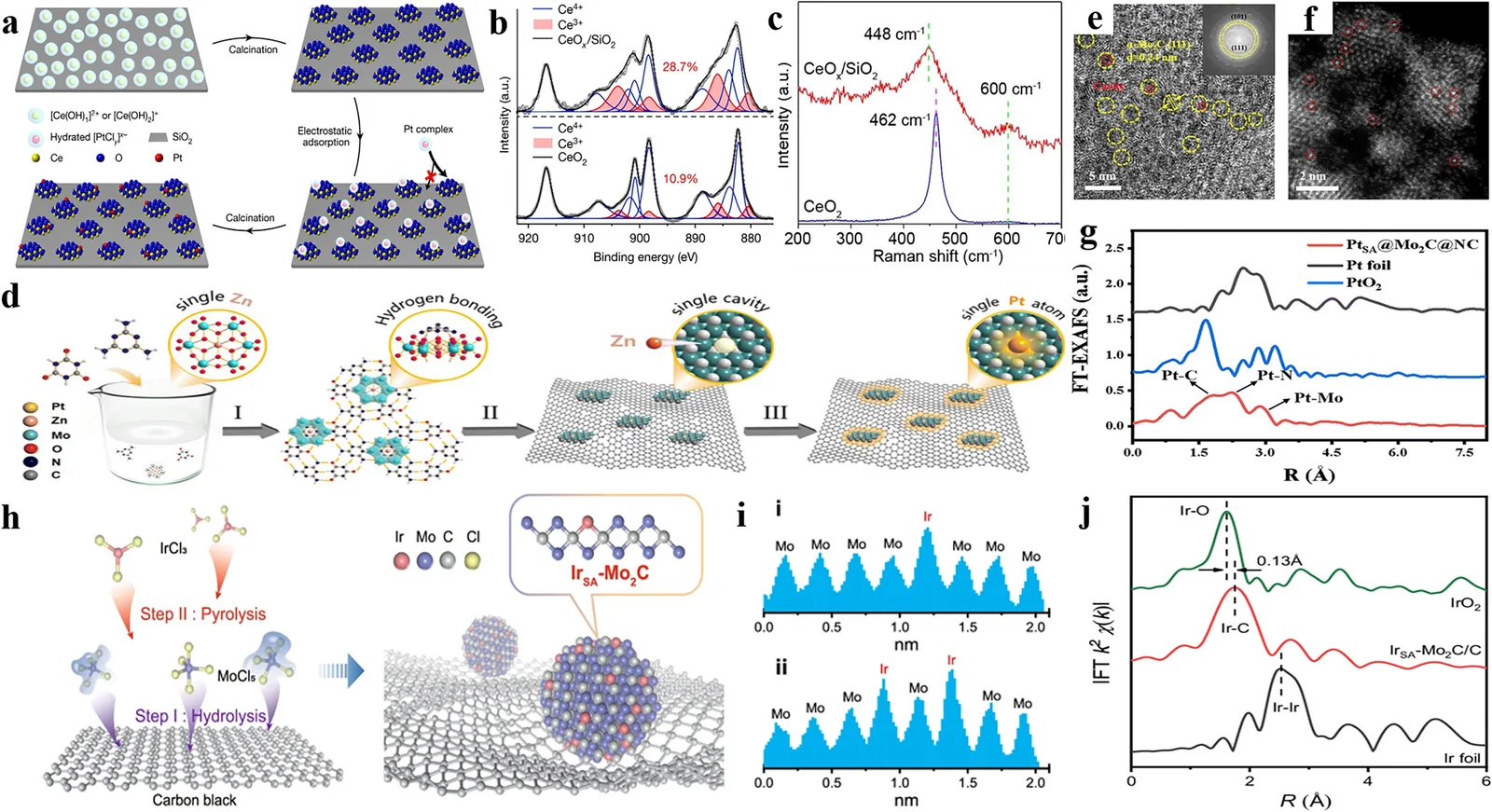
Two-step synthesis of SANIs and their characterization techniques. **a** Preparation process of functional CeO_*x*_ nanoglue islands and the preparation process of CeO_*x*_/SiO_2_-supported Pt_1_ single-atom catalysts. **b** Ce 3*d* XPS data characterizing SiO_2_-supported CeO_*x*_ nanoclusters (top) and pure CeO_2_ powders (bottom). **c** Normalized Raman spectra of the as-synthesized CeO_*x*_/SiO_2_ and pure CeO_2_. Comparison of cell volume and magnetic moment of three configurations. Reproduced with permission[34], Copyright 2022 Springer Nature. **d** Synthesis of Pt_SA_@Mo_2_C@NC (I: self-assembly, II: 5 h, N_2_ at 800 °C, and III: Pt^4+^ loading). **e** HRTEM and **f** images of PtSA@Mo_2_C@NC. **g** FT curves of PtSA@Mo_2_C@NC, PtO_2_ and Pt foil. Reproduced with permission [51], Copyright 2024 Royal Society of Chemistry. **h** Schematic illustration of the synthesis procedure. **i** Intensity profiles of the line i and ii in AC-HAADF-STEM image. **j** Corresponding FT *k*^2^-weighted EXAFS spectra. Reproduced with permission [44], Copyright 2024 Springer Nature

To achieve selective deposition of Pt SACs on CeO_*x*_ nano-islands, Li et al. utilized pH-regulated electrostatic interactions to direct the negatively charged Pt precursors to preferentially anchor on the positively charged CeO_*x*_/SiO_2_ rather than on the negatively charged SiO_2_ support (Pt/CeO_*x*_/SiO_2_) (Fig. 4a) [34]. CeO_*x*_/SiO_2_ exhibited a higher Ce^3^⁺ content (Fig. 4b) and defect density (Fig. 4c) compared to CeO_2_ and CeO_2_ NPs/SiO_2_, which ensured its strong anchoring of Pt atoms even at high temperatures. It was noteworthy that Pt NPs larger than 1 nm in size were not generated even at high Pt loading (4 wt%) and high temperatures, highlighting the effectiveness of the CeO_*x*_ nanoglue strategy in stabilizing the localization of Pt species and preventing their aggregation. When a high loading of Pt SACs was required, this could be accomplished by increasing the surface area of SiO_2_/density of CeO_*x*_ nanoclusters, demonstrating the scalability of this strategy for industrial applications.

The successful synthesis of Pt/CeO_*x*_/SiO_2_ opens a new era of SACs and paves the way for the study of SANIs. Considering that the defects within the paired nano-islands could also serve as anchor sites for atomic dispersion, the “defect substitution” strategy was proposed for the preparation of PtSA@Mo_2_C@NC (Fig. 4d) [51]. This strategy generated ultrafine Mo_2_C enriched with Zn defects by pyrolyzing ZnMo_6_, and the Pt SACs were precisely anchored and confined within the defects of the Mo_2_C nano-islands (Fig. 4e, f), achieving precise “defect substitution” at the atomic level. The Pt SACs generated in PtSA@Mo_2_C@NC predominantly employed axial N coordination (MoCPt–N) (Fig. 4g). This N coordination acted like a “safety belt” that stabilized the axially anchored Pt SACs while serving as a key transit station for accelerated electron transport. Notably, the introduction of Pt SACs also promoted the generation of thermodynamically stable *α*-Mo_2_C rather than metastable *β*-Mo_2_C. This strategy was successfully extended to the Ru and Ir systems, providing a generalized platform for the design of precisely spatially confined SANIs.

It is worth mentioning that Mo_2_C possess a Pt-like electronic structure, which predestines its unique binding properties as a nano-island with guest Ir SACs [78, 79]. In the report of Fang., Ir was deposited on carbon black by impregnation to grow MoO_*x*_, accompanied by high-temperature treatment C would diffuse from the carbon black into MoO_*x*_, and Ir SACs were then dispersed on Mo_2_C NPs to form IrSAMo_2_C/C catalysts depending on the potential (Fig. 4h) [44]. Unlike the “defect substitution” mechanism, the Ir SACs in this system occupied the substituted Mo sites in the hexagonal Mo_2_C lattice (Fig. 4i), forming a stable Ir-C coordination bond (Fig. 4j) with coordination parameters very close to those of the native Mo-C bond. This site-specific substitution strategy took advantage of the nearly identical bond lengths and coordination environments between the host and guest atoms to ensure the excellent dispersion stability of Ir SACs on the Mo_2_C nanolattice. Both approaches highlight the critical role of defect engineering and coordination chemistry on nano-islands in stabilizing the SACs conformation.

Currently, two synthesis strategies, one-step and two-step methods, are mainly used for the fabrication of SANIs. The one-step method is simple and relies on electron- support interactions, lattice confinement, or non-metallic coordination to stabilize SACs. Impregnation-pyrolysis is the predominant one-step technique, but the high energy consumption and hours-long high-temperature treatment have hindered the diffusion of this technology. Emerging electrical pulse and microwave-assisted methods overcome these limitations and enable rapid synthesis of SANIs, but they have more stringent support requirements (conductivity/polarity). Notably, the order of sequential addition of the two supports and the effect of the size of the formed nano-island particles need to be considered in these methods. The one-step hydrothermal synthesis method simplifies the fabrication of SANIs while optimizing the key parameters for the sequential addition of the two supports. Since the one-step method has limitations in controlling metalprecursor interactions and achieving precise loading of SACs. This has driven the strategic rise of the two-step synthesis. In the two-step method, nano-islands induce electrostatic interactions, defect substitution and coordination environments to meet precise SACs localization and stabilization. The research process of the two-step synthesis is more focused on exploring the localization accuracy of SACs precursors on nano-islands. However, the two-step synthesis also has limitations, including uncontrollable metalsupport coordination environments, the risk of structural collapse caused by thermal activation processes, and stringent requirements for support pore structure and thermal stability. In the future, in the preparation of SANIs, it can start from overcoming the strict requirements of the supports (e.g., electrical conductivity of electric pulse and polarity of microwave) to develop hybrid supports compatible with electric and microwave fields. Meanwhile, precisely adjusting the size, density, and spatial distribution of nano-islands for tailored functionality is another idea. Or combine density functional theory (DFT) with multi-scale simulations to predict optimal supportprecursor combinations.

The application of advanced characterization techniques is crucial for a more accurate understanding of the composition, structure, and chemical state of SANIs. Table 2 summarizes the current applications and limitations of commonly used static and dynamic characterization techniques for SANIs. The combined application of characterization techniques can not only provide guidance for the synthesis process of catalysts, but also support a deeper understanding of the structurestabilityactivity relationship. In addition, the application of dynamic characterization provides important research tools for exploring reaction mechanisms and the evolution and deactivation mechanisms of active sites.

**Table 2:** Applicability and limitations of various characterization techniques in the structural analysis

SACs various characterization techniques	Applicability	Limitations
HAADF-STEM (High-angle annular dark-field scanning transmission electron microscopy)	HAADF-STEM has an ultra-high resolution of 0.1 nm, enabling the distribution of SA sites to be observed with the naked eye, especially when the number of atoms in SACs is significantly higher than that in the substrate	HAADF-STEM can only provide information on the morphology and dispersion state of metal sites, but can not identify their composition. Therefore, it is difficult to completely confirm SAs using STEM alone. In this case, it is necessary to combine STEM with energy dispersive X-ray spectroscopy (EDS)
XAFS (X-ray absorption fine structure)	1. XANES spectroscopy measures X-ray absorption in the energy range from 30 to 50 eV of the absorption edge, and is mainly used to determine the oxidation state and nearest-neighbor coordination of atoms. 2. EXAFS spectroscopy measures X-ray absorption in the energy range from 50 to 1000 eV or higher, and is mainly used to determine the chemical state and coordination information of elements	1. The structure of metal SACs is not directly determined by EXAFS. Instead, the measurement data needs to be fitted to a structural model, which largely depends on subjective interpretations of statistical validity and distinctions from other structures. 2. EXAFS is an averaging technique, and the parameters obtained from the fitting represent the average values of all different types of elements present in the sample
XPS (X-ray photoelectron spectroscopy)	XPS is a widely used characterization technique that can detect the chemical and compositional properties of solid surfaces. Due to its depth of information, typically within 1 nm, XPS is particularly well suited for identifying the surface state of SACs in compact (planar) samples	1. XPS is limited to detecting surface species and may not provide sufficient information for SACs with low loading amounts. 2. Like other X-ray-related technologies, XPS may cause material changes due to exposure to X-ray beams
In situ Raman Spectroscopy	1. In situ Raman spectroscopy can be used to track in real-time the coordination adjustments, migration, and aggregation of SAs under reaction conditions. 2. It can be used to identify adsorbed species and reaction intermediates. 3. It can be used to observe the activation process and deactivation mechanism of SACs under reaction conditions	1. In situ Raman spectroscopy has weak signals and is insensitive to the vibration modes of light elements (such as H and Li). 2. Some supports will produce fluorescence to mask the Raman signal. 3. High temperature and high pressure conditions have high requirements for instrument sealing and optical window materials, and it is difficult to capture millisecond level rapid dynamic processes
DRIFTS (Diffuse reflectance infrared fourier transform spectroscopy)	DRIFTS provides information about the properties of the catalyst surface by detecting the interaction between probe molecules and the catalyst surface	1. DRIFTS relies on the interaction between CO and surface adsorption sites, so it cannot detect SA sites that do not bind with CO, or SAs embedded in bulk phases. 2. The signal strength and resolution of DRIFTS may be affected by the surface properties of the sample, such as surface roughness and chemical composition
NMR (Nuclear magnetic resonance)	NMR can be used to characterize the environment of catalytic centers and determine the coordination environment and binding sites of metal atoms. NMR is suitable for studying the dynamic behavior of molecules on time scales ranging from seconds to milliseconds, such as molecular rotation and conformational exchange	1. Limited to specific situations where the material must exhibit sufficient magnetic response. 2. The resolution and sensitivity of solid-state NMR are usually lower, and the equipment is complex. 3. NMR may not effectively capture dynamic processes that are faster (such as picosecond molecular vibrations) or slower (such as hourly enzyme catalyzed reactions)
EPR (Electron paramagnetic resonance)	EPR is highly sensitive to unpaired electrons and can provide information about the electronic environment and symmetry around the location of electron spin activity. The time resolution of traditional EPR is usually in the second range	1. The sample preparation process (such as grinding and pressing) may alter the dispersion state or coordination environment of single atoms, resulting in signal distortion. 2. Insufficient time resolution (e.g., capturing signals of instantaneous adsorption and charge transfer of SAs), and high-temperature, high-pressure, or corrosive gas conditions may damage the EPR cavity or affect microwave transmission

## Stability of SANIs: Deactivation Origins and Mitigation Strategies

SACs, with their exceptional atomic utilization efficiency and high catalytic activity, have become a research hotspot in multiple directions within the field of catalysis. However, their insufficient long-term stability remains a core bottleneck constraining their practical application. The integration of SACs with nano-islands structures in SANIs technology effectively addresses this stability issue. To deeply analyze the source of SANI’s high stability, this chapter will discuss the deactivation mechanisms of SACs in different application scenarios and their mitigation strategies based on the configuration of nano-islands. The aim is to provide theoretical guidance for designing highly stable SANIs and promoting their industrialization.

### SACs Migration and Aggregation

Thermocatalytic reactions require heating of the system to overcome activation energy barriers, encompassing various types of reactions (oxidation, reduction, and other reactions) [80, 81]. The development of highly stable thermal catalysts suitable for high-temperature oxidative environments is crucial for addressing energy and environmental challenges [82, 83]. Traditional supported metal catalysts are prone to deactivation due to sintering and aggregation of nanoparticles under harsh conditions. SACs also face the issue of sintering-induced deactivation caused by thermal diffusion-induced atomic aggregation [84, 85], especially in reducing catalytic atmospheres, the breaking of M-metallic bonds in SACs can trigger the formation of M-M bonds [86, 87]. To address this bottleneck, SANIs employ a “one-island-one-atom” architecture to achieve a “moving but not aggregation” mechanism, serving as a breakthrough solution to the activity-stability trade-off. Pioneering research in 2021 validated this concept by hosting Pt atoms (Pt/CeO_*x*_/SiO_2_) using defective CeO_*x*_ nano-islands [34]. The positively charged CeO_*x*_ possessed a stronger affinity (electrostatic interaction) for negatively charged Pt atoms than SiO_2_, ensuring that Pt SACs could move but remain within their active range. Extended X-ray absorption fine structure (EXAFS) spectra confirmed the atomic-level dispersion of Pt in the 0.4 wt% Pt/CeO_*x*_/SiO_2_ system, with no detection of Pt–Pt scattering paths. The simultaneously observed P-O coordination shell (coordination number was 4.5 ± 0.5) directly proved the bonding between Pt atoms and CeO_*x*_. Notably, even when exposed to an H_2_ environment at temperatures ranging from 400 to 600 °C, Pt atoms remained site-isolated, overcoming the atmospheric limitations of SACs and opening new avenues for designing highly robust thermal catalysts, thereby sparking a research boom in stable SACs based on nano-islands.

As research continues to advance, the SANIs system has achieved efficient dispersion and stabilization of SACs in electro-/thermalcatalytic through the stabilizing effect of nano-islands. In addition to electrostatic interactions, nano-islands primarily stabilize SACs through defect anchoring and electronmetalsupport interaction (EMSI) mechanisms. Taking the “one-island-one-atom” PtSA@Mo_2_C@NC system constructed via EMSI as an example, the Mo_2_C nano-island served as the primary support, suppressing Pt atoms migration through strong EMSI effects (Mo-Pt bonds) [51]. While the NC acted as the secondary supports, forming a 3D confined structure via axial Pt–N coordination (coordination number = 1.4), effectively restricting Pt atoms migration and maintaining sites isolation during electrochemical cycling. This enhanced EMSI effect originated from vacancy defects induced by Zn atoms depletion, which further induced axial pyridine N coordination to synergistically construct a stable structure. For the “one-island-multi-atom” SANIs configuration, the dispersion of multiple SAs sites on the nano-islands could also be achieved by providing abundant defect sites on the nano-islands to anchor the SAs [53].

**Fig. 5:**
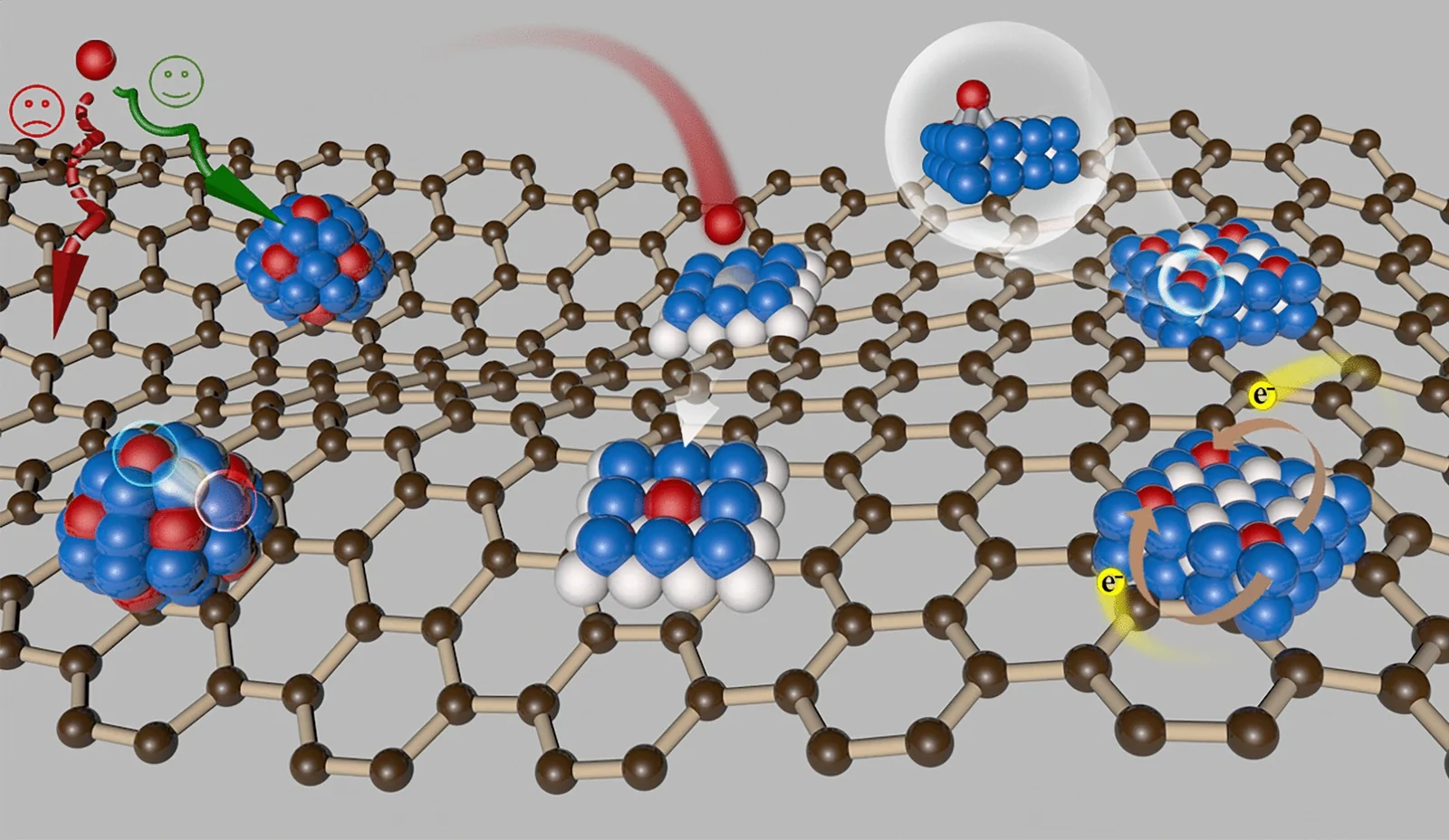
Excellent dispersion and stability of SANIs

As the core stabilizing unit of the SANIs system, nano-islands provide dual protection for SACs through their isolated, dispersed structure. On one hand, the high-density nano-islands dispersed on the support provide abundant dispersion sites for SACs. On the other hand, the rigid spatial barrier effectively isolates adjacent sites, completely blocking SACs migration and aggregation pathways. Currently, nano-islands stabilize SACs through three synergistic mechanisms: electrostatic interactions, defect site occupation, and EMSIs (Fig. 5).

### SACs Poisoning

**Fig. 6:**
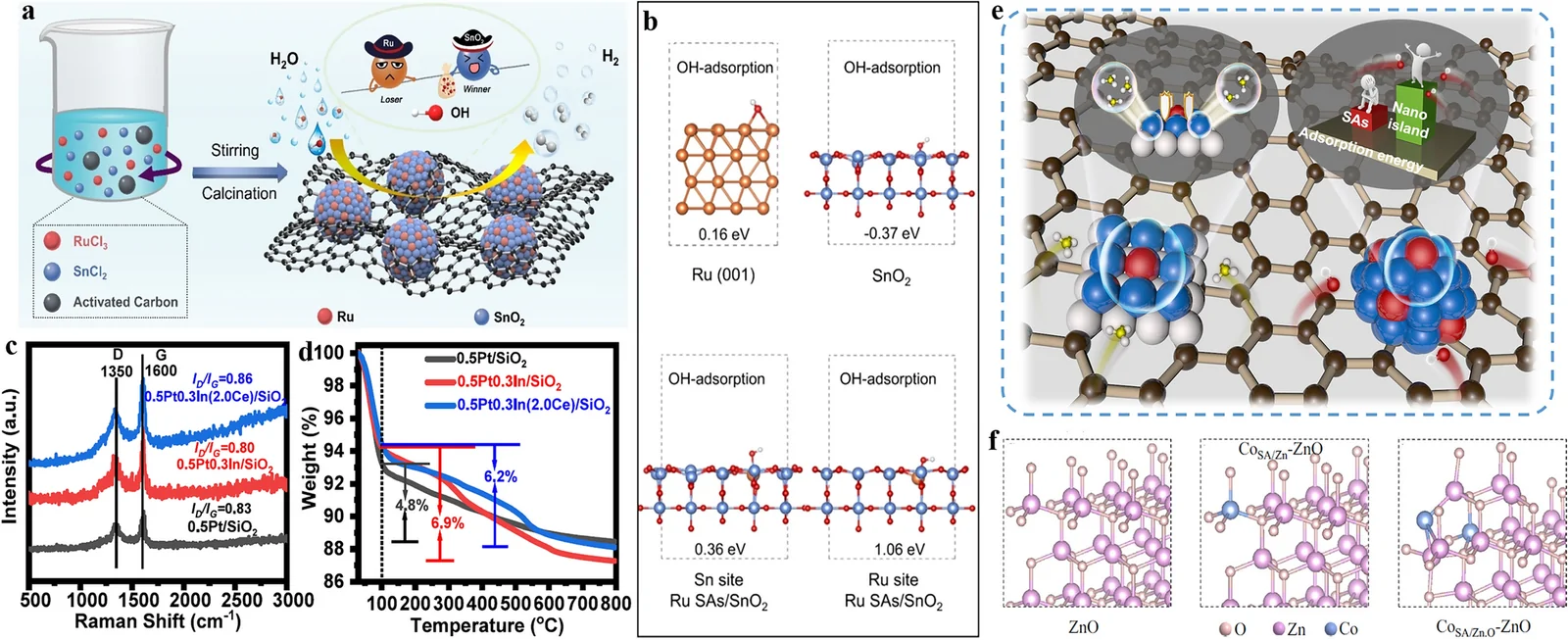
**a** Synthesis of Ru SAs-SnO_2_/C. **b** Ru (001), Ru site on Ru SAs/SnO_2_ and Sn site on Ru SAs/SnO_2_, and corresponding binding energies. Reprinted with permission [48], Copyright 2022 Wiley. **c** Raman spectra and **d** TG profiles of the catalysts. Reproduced with permission [56], Copyright 2024 American Chemical Society. **e** Site protection methods. **f** Three configurations of ZnO, Co replaced Zn and Co instead of Zn, O. Reprinted with permission [35], Copyright 2025 Springer Nature

Protecting the active sites of SACs is the key to realizing their theoretical advantages (high efficiency, superior selectivity, and low dosage). However, the exceptionally high surface free energy of SACs renders them prone to strong chemisorption with specific reactants/intermediates, leading to the formation of persistent overlayers that induce site poisoning [88, 89]. A typical example is sites poisoning caused by OH_ad_ species in alkaline electrocatalytic hydrogen evolution reactions (HER), which significantly reduces catalytic performance. To overcome this bottleneck, a “one-island-multi-atom” configuration of Ru SAs on SnO_2_/C nano-islands was constructed to induce a competitive adsorption mechanism (Fig. 6a) [48]. The SnO_2_/C component, which had pro-oxidative properties, preferentially adsorbed part of the OH_ad_ due to its high affinity for oxygen intermediates, alleviating the excessive binding of Ru sites. Its unique electronic structure also guided OH_ad_ to migrate toward the Ru active centers in a directed manner (Fig. 6b). The application of the competitive strategy regulated the adsorption of Ru SACs with reactants to enhance Ru sites toxicity resistance, with no decay in the polarization curves after 3,000 cycles, while Pt/C current density decayed by 35%. Additionally, it constructed an efficient electron transfer pathway (Ru to O) to support subsequent catalytic reactions.

In high-temperature dehydrogenation reactions, carbon deposition caused by the coupling of thermodynamically driven deep dehydrogenation with catalyst surface characteristics (e.g., metal agglomeration and excessive acidity) is a key challenge in protecting the integrity of active sites [90, 91]. Interestingly, the synergistic interaction between InCeO_*x*_ nano-islands and Pt SACs on SiO_2_ supports in the “one-island-one-atom” configuration effectively solved the problems of In^0^ generation and Pt sites blockage by carbon deposition [56]. On the one hand, the introduction of Ce could inhibit the generation of Pt-In bonds, thus suppressing the emergence of In^0^ species and reducing their coverage of Pt active sites (Fig. 6c). Concurrently, InCeO_*x*_ nano-islands acted as spatial physical barriers and appropriate MSIs effectively confined the isolated Pt^δ+^ sites, ensuring their high dispersion and structural integrity. On the other hand, InCeO_*x*_ nano-islands regulated the adsorptiondesorption behavior of propylene on Pt SACs, significantly weakening the adsorption strength of carbon deposition precursors. This design reduced carbon deposition to 6.2% (Fig. 6d) after 20 h of reaction at 550 °C, a 10% reduction compared to the PtIn/SiO_2_ system (6.9%). This synergistic protective mechanism endowed the catalysts with exceptional regeneration capability, enabling propylene yield to fully recover to fresh catalyst levels after O_2_ combustion treatment. Overall, the nano-islands allow for effective protection of the active sites through two pathways: competitive adsorption and prevention of carbon deposition (Fig. 6e). Both pathways possess the ability to address the problem at the source and slow down catalyst deactivation, thereby extending operational durability without compromising performance.

### SACs Dissolution

In the deactivation mechanism of SACs, the primary cause of metal dissolution is the acidic environment of the reaction system or the erosion by highly reactive oxygen species (ROS) [92–94]. In the peroxymonosulfate-based advanced oxidation process (PMS-AOPs), the dissolution of PMS leads to acidic conditions, which together with ROS attacks, cause the dissolution of active sites and performance degradation. The “island-sea synergistic” configuration in the SANIs structure overcomes this bottleneck through synergistic structural design. Nanoscale ZnO islands served as anchoring points, utilizing the similarity in ionic radii between Co^2+^ and Zn^2+^ to achieve lattice substitution doping, forming Co–O/CoZn coordination bonds that confined Co SAs within a stable structure to inhibit dissolution (Fig. 6f) [35]. Meanwhile, the large-area ZnO “Sea” substrate leveraged the amphoteric oxide properties to dynamically maintain the reaction microenvironment at a near-neutral pH value (~ 6.1, regardless of initial acidity or alkalinity), eliminating acid-induced dissolution risks at the source. This island-sea synergistic system kept Co leaching below the detection limit, maintaining 90% pollutant degradation efficiency after 10 cycles, successfully resolving the traditional trade-off between activity and stability. Furthermore, for acidic electrocatalytic HER systems, Pt SAs employed a hierarchical stabilization strategy to construct axial Ncoordination protection, effectively inhibiting Pt atoms leaching in acidic electrolytes, achieving a balance between catalytic activity and structural integrity [53].

SANIs effectively address the deactivation challenge of SACs through multi-level stabilization mechanisms. Their configuration design, tailored for different application scenarios such as thermal catalysis, electrocatalysis, and environmental catalysis, integrates spatial confinement, electronic regulation, and dynamic protection to provide a universal solution for the long-term stable operation of catalysts. Future research should focus on the application of different nano-islands configurations in complex systems, and further elucidate the universality and failure boundaries of protective mechanisms under acidic/alkaline media, high-temperature/high-pressure conditions, and multi-component coexistence environments during reaction processes.

## Bright Sparks within Nano-Islands and SACs

The high activity of SACs stems from their unsaturated coordination environment, while their stability is constrained by the strength of the binding energy between metal sites and the support, creating a natural trade-off [4, 95]. Notably, SANIs break limitation through the unique “moving but not aggregation” design concept of nano-islands, with their high stability having been thoroughly analyzed in the previous chapter. To elucidate the microscopic essence of this breakthrough mechanism, this section focuses on the regulatory mechanisms of activity by different types of SANIs. On one hand, SACs serve as the primary active sites, and nano-islands optimize the catalytic process through electronic structure modulation. On the other hand, nano-islands themselves also function as extended active sites directly participating in reactions, forming a synergistic dual-functional system with SACs sites.

### Flexible SACs Electronic Structure Modulation

**Fig. 7:**
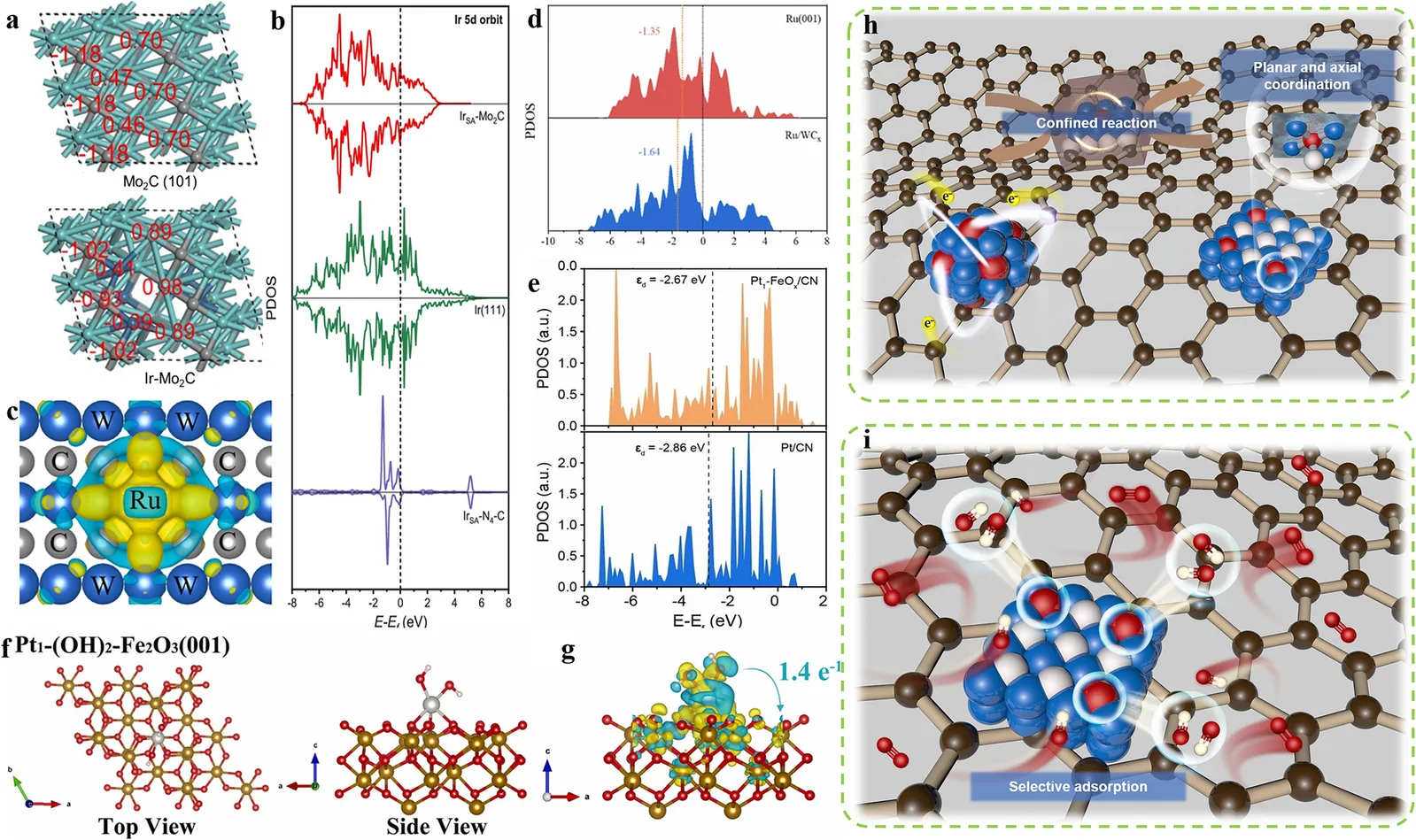
**a** Bader charge of the surface atoms of Mo_2_C (101) and IrSAMo_2_C. **b** PDOS diagram for *d* orbital of metals in IrSA-Mo_2_C, Ir(111) and IrSAN_4_C. Reproduced with permission [44], Copyright 2024 Springer Nature. **c** Differences in charge density over Ru SCs under lattice constraints. **d** d-band of Ru in different samples. Reproduced with permission [45], Copyright 2024 Elsevier. **e** Local density of states and d-band centers over Pt_1_FeO_*x*_/CN and Pt/CN samples. Reproduced with permission [53], Copyright 2022 Wiley. **f** Optimized structures of Pt_1_(OH)_2_Fe_2_O_3_(001). **g** Differential charge density over Pt_1_(OH)_2_Fe_2_O_3_(001). Reprinted with permission [55], Copyright 2023 Elsevier. **h** Electronic structure modulation within SANIs. **i** Selective enhancement within SANIs

The electronic regulation of nano-islands precisely tailors the electronic structure of SACs through charge transfer effects, serving as a critical link between atomic-scale active sites and macroscopic performance. Taking the constructed “one-island-one-atom” IrSAMo_2_C/C system as an example, the Mo_2_C nano-islands on the carbon supports to induce electron transfer, effectively regulating the electronic states of Ir SACs [44]. Bader charge analysis (Fig. 7a) showed that Mo atoms on the Mo_2_C surface transferred electrons to adjacent Ir atoms, resulting in highly delocalized partial density of states (PDOS) of the Ir-5d orbitals in IrSAMo_2_C (Fig. 7b), exhibiting metal-like 5*d* orbital characteristics similar to those of Ir(111). This electronic engineering significantly optimized the binding properties of Ir sites toward reaction intermediates, enabling the directional growth of Ir SACs with delocalized electronic configurations that successfully reconciled the inherent trade-off between catalytic performance and stability. Interestingly, recent studies have further revealed the structure–property relationship between nano-islands concentration and SACs distance. In the “one-island-multi-atom” configuration, O vacancies provided from CeO_2-*x*_ nano-islands acted as electronic traps to regulate Pt SAs density, achieving spatial structural control of Pt sites [59]. When the Pt–Pt distance was at the third-nearest-neighbor (3NN) distance, Pt SA transferred charge to the CeO_2-*x*_ surface, thereby enhancing intermediates adsorption and achieving optimal catalytic performance. This electronic restructuring exhibited a universal regulatory mechanism, where the crystal symmetry of the nano-island (e.g., the (111) crystal plane of CeO_2_) and defect concentration jointly determined the SACs distance, enhancing catalytic performance through charge redistribution.

The d-band center theory can elucidate the binding characteristics between reactants and the catalyst surface, and nano-islands can also regulate the d-band of SACs to optimize the adsorption/desorption balance of intermediates. The “one-island- multi-atom” configuration of Ru/WC_*x*_ was constructed by Chen et al., elucidating the d-band regulation of Ru SAs by WC_*x*_ nano-islands [45]. Ru atoms occupied W atoms on WC_*x*_ nano-islands to form shorter C-Ru bonds confined within the WC_*x*_ lattice, resulting in significant charge separation between Ru SAs and surrounding W atoms, with charge being distributed unevenly around Ru atoms (Fig. 7c). Compared to the Ru(001) surface, the d-band centers of lattice-confined Ru atoms were significantly shifted downward, away from the Fermi level (Fig. 7d), promoting desorption kinetics.

In advancing the development of electronic regulation mechanisms, in the “one-island- one-atom” configuration, FeO_x_ nano-islands on CN regulated the d-band center of Pt SAs to shift upward, achieving optimal matching of intermediate adsorption strength [53]. The regulation of the d-band originated from the FeO_x_ nano-islands acting as an electron donor on Pt SACs (Fig. 7e), leading to changes in the Pt-O bond length and coordination number within Pt SACs, which differed from the changes caused by Ru SAs replacing W atoms to form C-Ru bonds as described above. Interestingly, this study also investigated the influence of nano-islands size on the coordination environment of Pt SACs. Compared to Pt_1_-Fe_2_O_3_/CN containing Fe_2_O_3_ nanoparticles (15 nm), the Pt-O bond length (1.98 Å) and coordination number (4.3) of Pt in Pt_1_-FeO_x_/CN (1.07 nm) were significantly reduced by 2.5% and 20.4%, respectively, which further facilitated the achievement of optimal intermediates adsorption strength. It was worth noting that the axial N coordination mentioned in the stability section could also induce a downward shift of the d-band center in Pt SAs, reducing the number of occupied electrons in the Pt 5d orbitals and significantly enhancing the adsorption of intermediates [51].

It is particularly worth noting that orbital interactions between active sites and reactants have a decisive influence on catalytic performance, with their essence determined by precise regulation of the spin state of active sites. Beyond the d-band center theory, the combination of nano-islands and SACs has pioneered a new paradigm in spin engineering. Liang et al. revealed that in a “one-island-one-atom” configuration, FeO_*x*_ nano-islands induced charge-directed delocalization via Pt-O-Fe bonds (Fig. 7f), stabilizing Pt sites in a low-spin state (Fig. 7g) [55]. This electronic restructuring significantly enhanced selectivity, as low-spin Pt preferentially activated the C = O groups of cinnamaldehyde (CAL) rather than the C = C bond, achieving a breakthrough in hydrogenation selectivity.

The electronic structure regulation is achieved through a multi-scale synergistic strategy, including interfacial charge migration, microenvironment engineering, and spatial constraint effects (Fig. 7h), making the nano-islands a powerful tool for atomically precise control of electronic structure. Meanwhile, this mechanism can also selectively optimize specific group reaction pathways (Fig. 7i), providing a new dimension for the rational design of highly selective catalysts.

### Patulous SACs Sites Replenishment

**Fig. 8:**
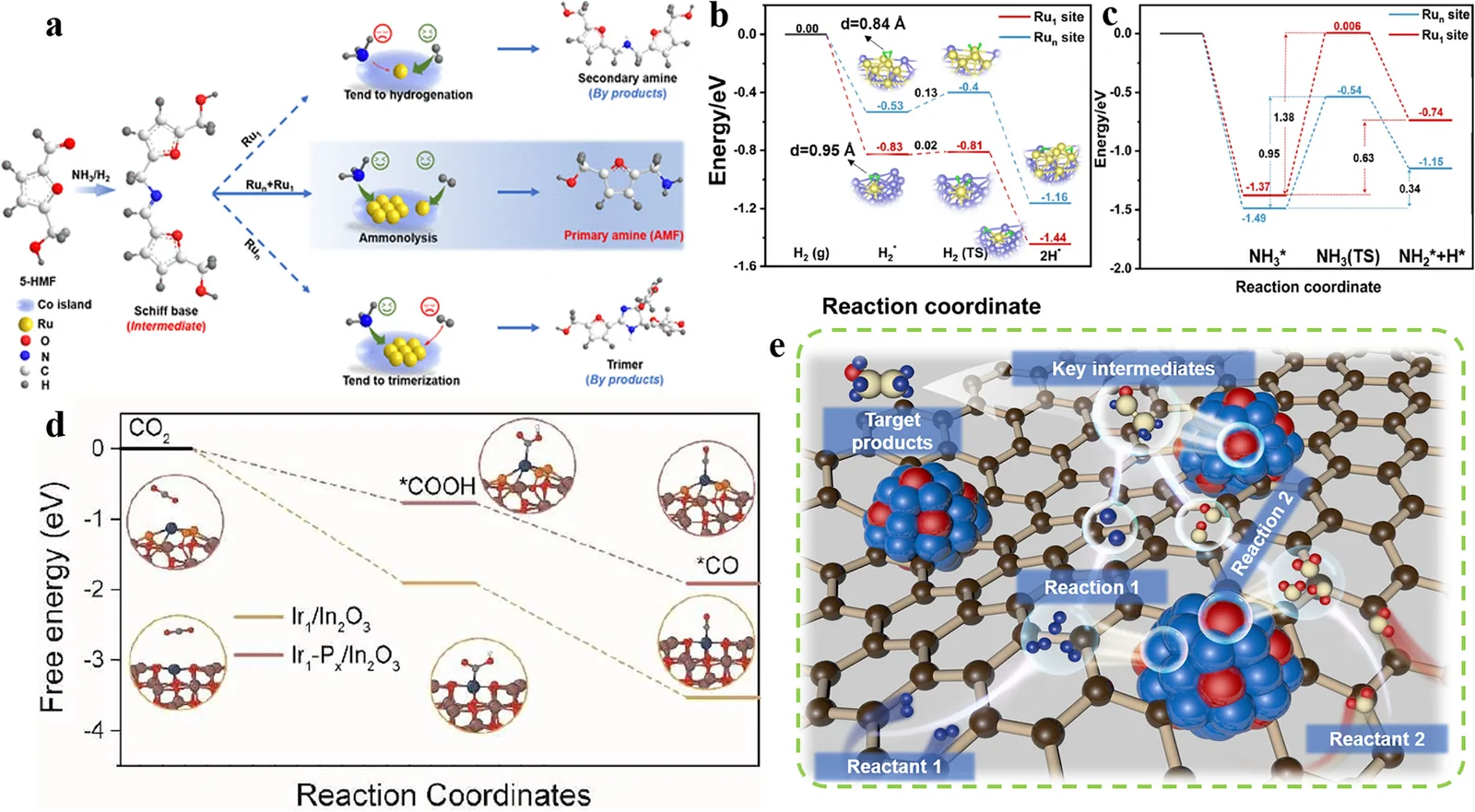
**a** Reaction behavior over H_2_ and NH_3_ in 5-HMF reduction amination reaction over the sites of Ru_1_ and Ru_*n*_. **b** Adsorption and desorption energy spectra of H_2_ at the sites of Ru_1_ and Ru_*n*_. **c** Adsorption and desorption energy spectra of NH_3_ to NH_2_* and H* on Ru_1_ and Ru_*n*_ sites. Reprinted with permission [46], Copyright 2024 American Chemical Society. **d** ΔG of CO_2_ reduction to CO at the Ir active site, along with the corresponding crystal structures of different samples as calculated by the GGA/PBE level. Reprinted with permission [57], Copyright 2025 Wiley. **e** Patulous SACs sites replenishment

The performance of SACs in complex multi-step reactions is often constrained by co-adsorption scaling relationships, while the SANIs dual-active-site system overcomes this limitation through synergistic effects. Tandem catalytic systems promise to address these limitations by enabling kinetic optimization of successive reaction steps through stepwise relay mechanisms [96, 97]. A confined cobalt nano-islands system constructed based on the tandem catalytic concept features Ru_*n*_ sites that preferentially adsorbed and desorbed NH_3_ via multi-site bonding to generate active NH_2_* species (Fig. 8a, -1.49 eV), while Ru_1_ specialized in H_2_ desorption to generate H* (Fig. 8b, -0.84 eV) [46]. This site-specific division of labor resolved the competitive adsorption issue between NH_3_ and H_2_, enabling local microdomains to achieve NH_2_*/H* coverage equilibrium (Fig. 8c). Concurrently, the Co nano-islands subsequently served dual functions: (i) their confined structures contributed to the rapid diffusion of the generated H* and NH_2_* intermediates; and (ii) these nanostructures mediated the subsequent reaction of the diffused intermediate with surface adsorbed imine species, ultimately driving the catalytic process. This “island-sea synergistic” configuration not only overcame the limitations of traditional SACs but also established an efficient tandem reaction pathway through spatial and temporal control of intermediate transferred.

Additionally, parallel catalysts broaden reaction pathways and enhance stability through the synergistic action of multiple active sites, enabling the production of specific products (ethanol) through CO_2_ hydrogenation [57]. This achievement was realized through the synergistic effect of P islands on In_2_O_3_ nanosheets and Ir SACs precisely deposited on the P islands. In the “one-island-one atom” SANIs configuration, Ir SACs were primarily responsible for the adsorption and activation of CO_2_ molecules as well as for the C–C coupling process. The P islands optimized their electronic structure and enhanced their activity through specific coordination. Not only that, but they were also responsible for the H_2_ dissociation process, generating H* and facilitating the C–C coupling process. DFT calculations indicated that the introduction of P islands significantly reduced the reaction energy barrier from 1.23 eV for Ir_1_/In_2_O_3_ to 0.72 eV for Ir_1_-P_x_/In_2_O_3_ in the rate-limiting step (RLS), while the Gibbs free energy barrier for the entire reaction pathway decreased from 0.73 to 0.65 eV (Fig. 8d). This significant reduction in the energy barrier stemmed from the multi-component synergistic system formed by P islands and Ir SACs, which optimized H* transferred and C–C coupling processes through spatially precise arrangement of complementary active sites, ultimately achieving an efficient catalytic process. The multicomponent system between SACs and nano-islands (tandem or parallel, Fig. 8e) demonstrates how the tailored spatial organization of complementary active sites can optimize complex reaction networks.

Overall, the synergistic interaction between SAC sites and nano-islands in SANIs is sufficient to regulate the electronic structure of the reaction sites (typically in a “one-island-one-atom” configuration), thereby enhancing intrinsic catalytic activity. Integrating two sites tailored for different catalytic reactions into the catalyst can generate a cascade catalytic effect, improving the efficiency of multi-step electrocatalytic reactions (typically in a “one-island-multi-atom” or “island-sea synergistic” configuration). Undoubtedly, the diverse configurations of SANIs offer an alternative strategy for constructing efficient and ideal catalysts suitable for various catalytic reactions.

## SACs Partner with Nano-Island to Open New Chapter in Catalysis

SACs herald a transformative era in catalysis through innovative nano-island structures that redefine atomic dispersion precision while enabling unprecedented stability and reactivity. This nano-island-mediated design paradigm not only revolutionizes and breaks the stability-activity trade-off, but also establishes a powerful framework for optimizing electronic interactions and maintaining catalytic performance under harsh operating conditions. Consequently, this chapter highlights the remarkable advancements of SANIs across diverse catalytic applications (Table 3). In particular, the fundamental mechanisms governing their superior stability, catalytic activity and reaction kinetics are used as a bridge as an analytical framework to link structural to performance optimization.

**Table 3:** Summary of SANIs performance in various catalytic applications

Catalysts	Synthesis Methods	Reaction	Performance	Refs
Ru/WC_*x*_	One-step	HER	1. Activity: Mass activity: 6000 mA mg^−1^; Turnover frequency: 3.89 H_2_^−1^ at -100 mV vs. RHE. 2. Stability: /	[45]
Ru_1+n_@Co/MMO	Two-step	Chemical synthesis	1. Activity: AMF production rate: 295 g g_Ru_^−1^ h^−1^. 2. Stability: 5 cycles	[46]
Pt_1_/SnO_2_/UiO-66-NH_2_	One-step	HER	1. Activity: Hydrogen evolution rate: 2167 µmol g^–1^ h^–1^. 2. Stability: 3 cycles	[47]
Ru SACs-SnO_2_/C	One-step	HER	1. Activity: Overpotential: 10 mV at 10 mA cm^−2^; Tafel slope: 25 mV dec^−1^. 2. Stability: 3000 cycles	[48]
Pt_SA_-MoS_2_/rGO	Two-step	HER	1. Activity: Overpotential: 11 mV at -10 mA cm^−2^ in 0.5 M H_2_SO_4_. 2. Stability: 10,000 cycles	[49]
Pt/CeO_*x*_/DMS	Two-step	CO conversion	1. Activity: T₉₀ of 143 °C for Pt/20 wt% CeO_*x*_/DMS, has the lowest Eₐ 2. Stability: 90% conversion at 147 ℃	[50]
Pt_SA_@Mo_2_C@NC	Two-step	HER	1. Activity: Mass activity: 75.21 A mg_Pt_^−1^ in 0.5 M H_2_SO_4_. 2. Stability: 12,000 cycles	[51]
Ir_SA_-Mo_2_C/C	Two-step	HER	1. Activity: Specific exchange current density: 4.1 mA cm^−2^_ECSA_. 2. Stability: 30,000 cycles	[44]
Pt_1_Mo_1_/Ni_3_S_2_	Two-step	HER	1. Activity: Overpotential: 53 mV at 10 mA cm^−2^; Tafel slope: 49.6 mV dec^−1^. 2. Stability: 60 h	[52]
Pt_1_FeO_*x*_/CN	One-step	ORR	1. Activity: Peak power density of 45.1 mW cm^−2^. 2. Stability: 120 h	[53]
PtCl_2_Au(111)/GDY	Two-step	Methanol and ethanol oxidation	1. Activity: Mass activity: 175.64 A mg_Pt_^−1^ (MORs) and 165.35 A mg_Pt_^−1^ (EORs). 2. Stability: 1000 cycles	[54]
Pt/CeO_*x*_/SiO_2_	Two-step	CO oxidation	1. Activity: H_2_ activation increases the CO oxidation rate by two orders of magnitude and decreases the apparent activation energy. 2. Stability: 4 cycles	[34]
Pt_1_-FeO_*x*_/SBA-15	Two-step	Selective hydrogenation	1. Activity: High selectivity to COL (> 95%). 2. Stability: 3 cycles	[55]
0.5Pt0.3In(yCe)	One-step	Propane dehydrogenation	1. Activity: 92.2% selectivity toward propylene, specific activity of 12.5 min^−1^, and a stable propane conversion of 67.1%. 2. Stability: 2 cycles	[56]
Ir_1_-P_*x*_/In_2_O_3_	Two-step	CO_2_ Hydrogenation	1. Activity: Ethanol yield: 3.33 mmog^−1^ h^−1^; TOF: 2108 h^−1^. 2. Stability: 5 cycles	[57]
Co_SA/ZnO_-ZnO	One-step	/	1. Activity: K = 98.2 min^−1^ M^−1^. 2. Stability: 5 cycles	[35]
Pt_1_@POMs@PC	Two-step	HER	1. Activity: Overpotential: 3.8–8.3 mV at 10 mA cm^−2^; Tafel slope: 9.76–18.5 mV dec^−1^; TOF of Pt₁@PW₁₂@PC at 0.03 V overpotential up to 24.9 s^−1^. 2. Stability: 100 h	[58]
Pt_SA_-M-CeO_2-*x*_ /rGO	One-step	HER	1. Activity: Overpotential: 25 mV at 0.5 M H₂SO₄, 33 mV at 1 M KOH, 21 mV at 1 M PBS; Tafel slope: Acidic conditions: 22.8 mV dec^−1^ at 0.5 M H_2_SO_4_, 57.9 mV dec^−1^ at 1 M KOH. 2. Stability: 90 h/2000 cycles	[59]
Pt-Cl/CeO_x_/SiO_2_	Two-step	CO oxidation	1. Activity: The apparent activation energies: 66.5 ± 1.9 kJ mol^−1^. 2. Stability: 4 cycles	[60]
Pt_1_/CeO_x_/SiO_2_	Two-step	CO oxidation	1. Activity: Achieving 90% COconversion at 96 °C. 2. Stability: /	[61]
Pt/CeO_x_/SiO_2_	Two-step	Ethylene hydrogenation	1. Activity: The TOF of the catalyst is 0.096 ± 0.004 s^−1^. 2. Stability: Maintained stability under harsh conditions in flowing H_2_	[62]

### Batteries

The energy density of commercial lithium-ion (Li-ion) batteries using Ni/Co-based cathodes and graphite anodes has encountered a bottleneck due to the limited specific capacity of the electrode active materials [98–100]. Fuel cells with ultra-high energy density are regarded as ideal candidates for future energy conversion systems. In recent years, many remarkable features of SANIs have led to their emergence in fuel cells. In this section, the outstanding performance of SANIs in different reactions (including oxygen reduction reaction (ORR), hydrogen oxidation reaction (HOR), and methanol/ethanol oxidation reactions) is discussed, with a focus on the synergistic mechanisms under different reaction mechanisms.

#### Oxygen Reduction Reaction

**Fig. 9:**
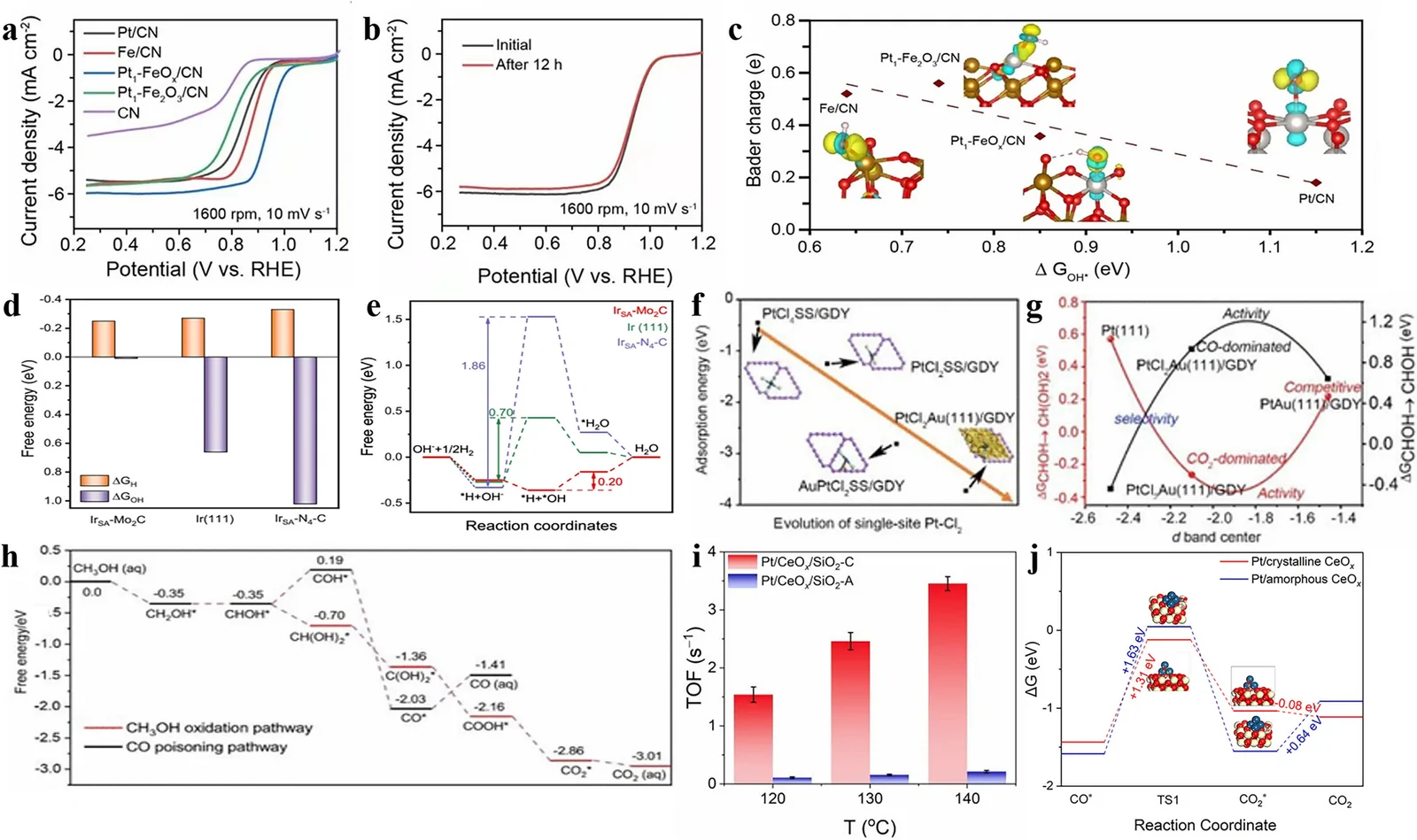
Applications of SANIs to batteries. **a** ORR polarization curves. Mechanism study of ORR process on the catalysts. **b** ORR polarization curves over Pt_1_FeO_*x*_/CN before and after the stability testing. **c** The relationship amongΔ*G*_OH*_ and Bader charge over different samples (Fe/CN, Pt_1_FeO_*x*_/CN, Pt/CN, and Pt_1_Fe_2_O_3_/CN). Reproduced with permission [53], Copyright 2022 Wiley. **d** The calculated adsorption Gibbs free energy of H and OH. **e** Gibbs free energy diagrams of HOR on the Ir_SA_Mo_2_C, Ir(111) and Ir_SA_N_4_C. Reproduced with permission [44], Copyright 2024 Springer Nature. **f** The variation of the adsorption energies of Pt-Cl_4_/Cl_2_ species in the studied samples with the evolution of individual PtCl_2_. **g** Volcano plot of PtCl_2_Au(111)/GDY showing the relationship between the activity and selectivity of MOR and the d band center position of Pt SAs (black line: CO poisoning process, red line: MOR process). **h** Study of the energy pathways of MOR and CO poisoning on PtCl_2_Au(111)/GDY (Reaction conditions: continuous solvent model with dielectric constant = 78.4, with a potential of 0.48 V vs RHE). Reproduced with permission [54], Copyright 2022 American Chemical Society. **i** Specific reaction rates expressed as TOF (per Pt active surface area) for Pt/CeO_x_/SiO_2_ catalysts. **j** CO oxidation proceeds on Pt/CeO_x_/SiO_2_ catalysts. Reproduced with permission [61], Copyright 2025 American Chemical Society

As a typical example, the “spark” between MO_*x*_ and Pt was investigated to develop efficient catalytic performance for ORR catalytic performance using CN as supports and different oxide clusters as nano-islands to disperse Pt atoms (Pt_1_-MO_*x*_/CN) [53]. Among the synthesized catalysts, Pt_1_-FeO_*x*_/CN tabulated to be the most promising catalyst (half-wave potential up to 0.94 V for RHE. (Fig. 9a) and outstanding stability (≈ 98% of the initial current density after 12 h of testing in alkaline medium (Fig. 9b). This breakthrough of simultaneously achieving high activity and stability stemmed from the dual functionality of FeO_*x*_. Structurally, FeO_*x*_/CN provided dispersed and independent sites that effectively stabilized and prevented the aggregation of Pt SACs. Electronically, FeO_*x*_ optimized the d-band center of Pt SACs by adjusting the bond energy of the Pt-O bond relative to Pt/CN. This electronic structure engineering optimized the adsorption strength of the OH* intermediates, as evidenced by the strong linear relationship between the OH* Bader charge and their corresponding free energies of adsorption (ΔG_OH*_) (Fig. 9c). Such precise regulation of the intermediate adsorption/desorption kinetics successfully circumvented the traditional activity-stability trade-off compromise in ORR catalysis. The unique structural configuration and unprecedented ORR performance of Pt₁-FeO_*x*_/CN enabled the zinc-air battery to operate under extreme conditions (-40 °C), delivering a peak power density of 45.1 mW cm^–2^ and maintaining stable cycling performance for 120 h.

#### Hydrogen Oxidation Reaction

In addition to the ORR occurring at the cathode, the hydroxide reaction HOR occurring at the anode is also a crucial half-reaction whose high efficiency ensures the stable operation of the anode [101, 102]. However, since the metal microenvironment of M-N_4_-C atomically dispersed catalysts is significantly different from that in pristine metal NPs, it may trigger inappropriate adsorption of HOR intermediates and result in unsatisfactory HOR performance [103, 104]. The key to the efficient realization of HOR is to regulate the microenvironment of the metal sites to meet the appropriate intermediate adsorption, and the introduction of nano-islands provides a driving force for the above regulation. In the work by Zhuang et al., Ir SACS anchored on Mo_2_C NP -supported carbon (IrSA-Mo_2_C/C) was engineered to straddle the obstruction problem in HOR and alkaline membrane fuel cells by skillfully utilizing the Pt-like energy band structure of Mo_2_C [44]. As shown in Fig. 9d, the Ir-5*d* orbitals of the IrSAMo_2_C exhibited a large degree of delocalization contrasted sharply with the delocalized d-orbitals of conventional IrN_4_C catalysts, a consequence of electron transfer from Mo to adjacent Ir sites. This electronic modulation on the one hand significantly enhanced its total density of states (DOS) at the Fermi level, leading to better electronic conductivity. On the other hand, the optimized H binding energies of Ir SACs was accompanied by a moderate increased in the OH binding energies (Fig. 9e), ultimately significantly accelerating the HOR reaction kinetics.

#### Methanol/ethanol Oxidation Reactions

Obviously, proper adsorption between the catalyst and the intermediate is important to enhance its catalytic performance. Excessive adsorption will disrupt the adsorptiondesorption equilibrium, leading to the occupation of active sites and reducing the reaction rate. Besides, intermediates (e.g., CO) with strong affinity can form strong chemical bonds with the catalyst, leading to poisoning of the active site, which has been an insurmountable bottleneck in the methanol oxidation reactions (MORs) and ethanol oxidation reactions (EORs) [105–108]. To address this bottleneck, Li et al. made a breakthrough by epitaxially growing Au(111) quantum dots (PtCl_2_Au(111)/GDY) integrated with PtCl_4_ species on graphdiyne (GDY) [54]. The catalysts showed unprecedented electrocatalytic activity with mass activities of 175.64 and 165.35 A mg_Pt_^–1^ for MOR and EOR, respectively, and maintained extremely high stability even after 110 and 60 h of operation. Such outstanding stability was due to the atomically dispersed Pt-Cl_2_ species and the stabilization effect of Au(111)/GDY (Fig. 9f). For its fantastic MOR and EOR activities were due to the introduction of Cl induced a negative shift of the d-band of Pt atoms, which weakened the adsorption affinity of Pt for CO and improved the catalytic performance. More importantly, the d-band center position also possessed an important effect on the activity and selectivity of MOR. Remarkably, PtCl_2_Au(111)/GDY system with a suitable d-band operated predominantly via the MOR pathway (Fig. 9g), as evidenced by the significant inhibition of CO-related intermediates (Fig. 9h). This work established a paradigm for the rational design of catalysts by utilizing electronic structure modulation to lift the limitations of activity and stability in alcohol oxidation systems.

While ligand selection is a dominant factor in the design of supported metal catalysts, its systematic influence on reactivity remains largely underexplored. To address this gap, Zhang et al. developed a chlorine ligand modulation strategy, tailoring the coordination environment of Pt SACs on SiO_2_supported CeO_x_ nano-islands (PtCl/CeO_x_/SiO_2_) to unravel the mechanistic impact of ligand engineering [60]. The Pt_1_Cl_2_O_4_ configuration formed in the PtCl/CeOx/SiO_2_ system demonstrated exceptional kinetic advantages. The energy barrier for adsorbed CO* oxidation via reaction with coordinatively unsaturated oxygen (0.45 eV) was significantly lower than that on conventional Pt_1_O_6_ configuration (0.89 eV). Moreover, the unique electronic structure of Pt_1_Cl_2_O_4_ lowered the oxygen vacancy formation energy to -0.08 eV, in stark contrast to the 1.80 eV required for Pt_1_O_6_, ensuring a sustainable supply of reactive oxygen species for CO oxidation. Consequently, the chlorine-modified catalyst achieved a CO oxidation turnover frequency (TOF) twice that of the chlorine-free counterpart in the 180–230 °C range, highlighting the effectiveness of ligand regulation. Given the support crystallinity plays a pivotal role in dictating catalytic performance, this team engineered CeO_*x*_ clusters on high-surface-area SiO_2_ and precisely modulated their crystallinity through controlled calcination [61]. When evaluated in the temperature range of 120–140 °C, the highly crystalline Pt/CeO_*x*_/SiO_2_C exhibited a 15-fold increase in specific reaction rate compared to its amorphous Pt/CeO_*x*_/SiO_2_A (Fig. 9i). This dramatic enhancement was ascribed to the superior reactivity of lattice oxygen in crystalline CeO_*x*_, which not only accelerated CO desorption kinetics but also reduced the temperature required for interfacial oxygen reduction. Attentionally, the rate-determining step for Pt/CeO_*x*_/SiO_2_ involved the reaction between adsorbed CO* and adjacent lattice oxygen to form CO_2_ and oxygen vacancies, rather than the dissociation of gaseous O_2_. The lower energy barrier for lattice oxygen participation in the initial transition state of Pt/CeO_*x*_/SiO_2_C provided a clear rationale for its exceptional activity (Fig. 9j).

### Hydrogen Evaluation Reduction

**Fig. 10:**
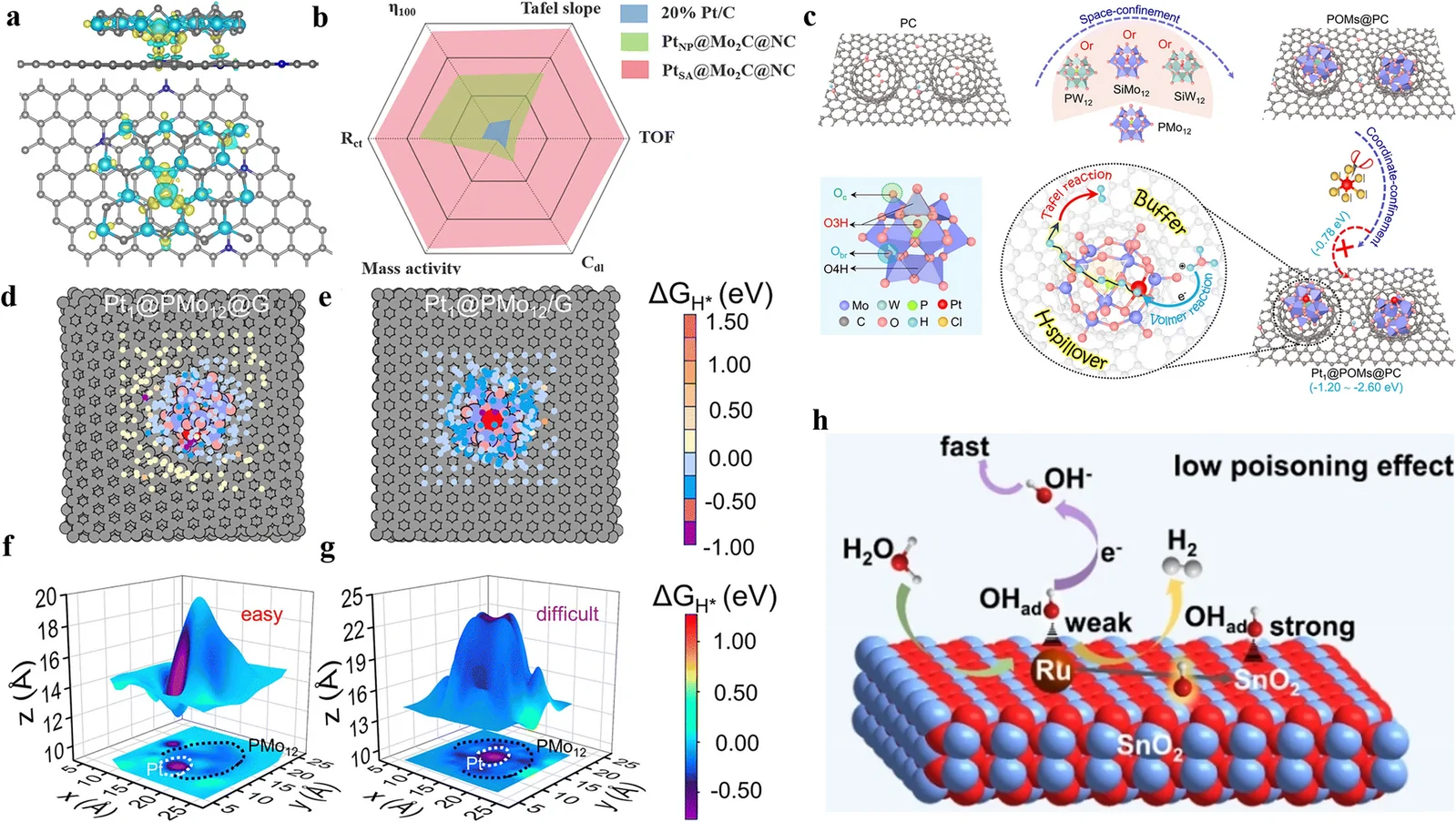
Applications of SANIs to HER. **a** Charge density differences over Pt_SA_@Mo_2_C@NC, the top figure showing the top view and the bottom figure showing the side view. **b** Percentage comparison of overpotential (η_100_), Tafel slope, TOF value, C_dl_ value, mass activity, and R_ct_ value over different samples. Reprinted with permission [51], Copyright 2024 The Royal Society of Chemistry. **c** Schematic diagram illustrating the fabrication processes of Pt1@POMs@PC. Reprinted with permission [58], Copyright 2024 The Royal Society of Chemistry. H adsorption free energy maps for HER over **d** Pt_1_@PMo_12_@G and **e** Pt-PMo_12_/G (model for non-confined PMo_12_ systems). 3D contour of ΔG_H*_ on **f** Pt_1_@POMs@G and **g** Pt_1_@POMs/G. Reprinted with permission [58], Copyright 2024 The Royal Society of Chemistry. **h** Intrinsic mechanism of improved HER activity over the sample of Ru SACs-SnO_2_/C. Reprinted with permission [48], Copyright 2022 Wiley

Clean energy production plays an irreplaceable and fundamental role in the development and progress of human civilization [109–111]. Hydrogen, an important support of clean energy, has an important position in reducing carbon emissions and promoting sustainable energy development [59]. The HER mechanism varies in different media (acidic and alkaline), yet it follows similar reaction steps. In proton-rich acidic media, the reaction proceeds through the Volmer step (H^+^  + e^–^ → H*), the Heyrovsky step (H* + H^+^  + e^–^ → H_2_), and the Tafel step (2H* → H_2_). In alkaline media, where protons originate from the dissociation of water molecules, the reaction first undergoes the Volmer step (H_2_O + e^–^ → H* + OH^–^) and then generates H_2_ via either the Heyrovsky step (H* + H_2_O + e^–^ → H_2_ + OH^–^) or the Tafel step, with water dissociation being the key process. Due to their outstanding catalytic performance and stability, SANIs have been applied to electrocatalytic HER for hydrogen production [49, 52]. Yue et al. utilized Pt SAs anchored in Mo_2_C islands and N-axially coordinated in the NC sea for HER in acidic and alkaline electrolytes [51]. Due to the EMSI of Mo_2_C nanoclusters to Pt SAs and the axial N coordination, the migration of electrons from Pt SAs towards NC, which contributed to the rapid electron transfer to the support during HER and optimized the electronic structure of the reaction sites (Fig. 10a). According to Gibbs free energy calculations, Pt_SA_@Mo_2_C@NC showed superior H_2_O dissociation, intermediates adsorption, and H_2_ desorption capabilities compared to Pt@Mo_2_C anchored only by Mo_2_C or Pt@NC with Pt–N coordination. Therefore, Pt_SA_@Mo_2_C@NC held unprecedented activity in electrocatalysis (Fig. 10b) and achieved a high mass activity of 75.21 A mg_Pt_^–1^ in 0.5 M H_2_SO_4_ and a H_2_ production of 6.3 mmol for 2 h with a 98.4% Faraday efficiency. Excitingly, the 3D coordination engineering, especially the axial coordination N, prevented the dissolution and migration of Pt SACs, conferring the long-term stability to Pt_SA_@Mo_2_C@NC even after 12,000 and 25,000 cycles in 0.5 M H_2_SO_4_ and 1.0 M KOH, respectively.

In the HER process, a hindrance is H accumulation resulted by slow H migration kinetics, which reduces the H escape efficiency. Yan et al. significantly alleviated the H migration barrier by constructing an H-buffer chain linking the H escape pathway from Pt to the support [58]. In the constructed H-buffered chain, the Keggin-structured polyoxometalates (POMs) was first confined to a unimodal super-empty porous carbon (PC) with sub-1-nm pores, and subsequently individual Pt atoms were stabilized at the O_4_H site of the POMs, where the diverse oxygen and Mo–O bonds of POMs together with the Pt_1_ and PC supports constructed the H-buffer chain (Pt → O_br_ → O3H → Mo/W → O_c_ → PC_sub-1-nm_) (Fig. 10c). Notably, the confined POMs played a crucial role in the H-buffer chain. Compared to non-confined POMs (Pt_1_@POMs/G), Pt_1_@POM@G had a progressively lower ΔG_H*_ from the Pt_1_ sites to the support (Fig. 10d-g). Specifically, H was strongly adsorbed on Pt_1_ but weakly adsorbed on POMs, where the binding energies of O_br_, O3H, M, and O_c_ to H in the confined POMs were almost zero, which facilitated H desorption.

In alkaline environments, excessive adsorption capacity may lead to the poisoning of metal active sites by OH intermediates. Ru, as a representative of the Pt group metals, shows great potential in the HER. However, the excessively high binding energy between its sites and OH groups can hinder the proton reduction process, leading to catalyst poisoning and activity decline. Fortunately, the poisoning effect of the Ru sites can be significantly alleviated by competitive OH-adsorption through the introduction of other sites with affinity for OH. Zhang et al. immobilized Ru SAs on SnO_2_ nano-islands and reduced the poisoning of Ru SAs using the intense oxophilic property of SnO_2_ [48]. Based on the results of cyclic voltammetry curves, CO oxidation experiments and zeta potential tests, the adsorption capacity of SnO_2_ for OH was proved to be significantly stronger than that of Ru. According to theoretical calculations, the binding energy of OH on Ru SAs (1.06 eV) was significantly weaker than that of SnO_2_ (0.36 eV), and thus OH would be preferentially adsorbed on Sn sites. Therefore, SnO_2_ significantly facilitated the OH_ad_ transfer process and promoted the regeneration of Ru SACs, which effectively reduced the poisoning effect and enhanced the H_2_O dissociation ability of Ru active sites (Fig. 10h).

### Chemical Synthesis

Chemical synthesis, serving as the fundamental pillar of modern industrialization, exerts multi-dimensional impacts spanning: (i) frontier scientific discovery; (ii) industrial manufacturing paradigms; and (iii) sustainable societal evolution [112, 113]. The advent of SANIs represents a paradigm-altering innovation in precision catalysis. These atomically precise architectures unlock unprecedented avenues for manipulating reaction coordinates through quantum-confined active sites, while enabling sustainable synthesis protocols for energy-critical molecules and environmentally benign chemical transformations.

**Fig. 11:**
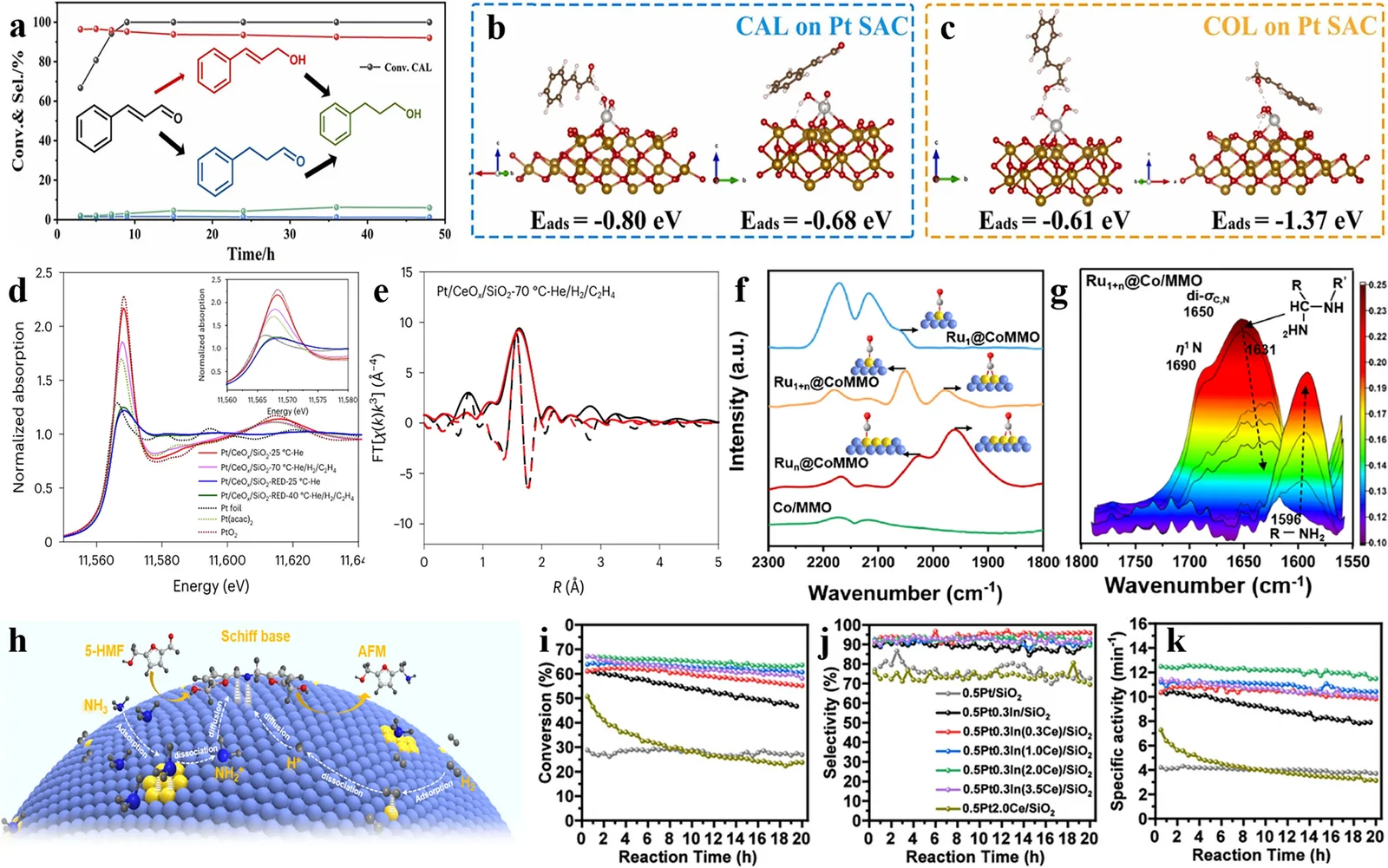
Leveraging SANIs in chemical synthesis. **a** Catalytic results for CAL hydrogenation over 1%Pt_1_-FeO_*x*_/SBA-15 at various reaction time. (Reaction conditions: 0.02 g catalyst; 25 mmol CAL; 3.0 MPa initial H_2_ pressure; 5 mL i-PrOH; 600 rpm and room temperature). **b** CAL adsorption over Pt_1_(OH)_2_Fe_2_O_3_(001) surface via C = O and C = C bonds. **c** COL adsorption over Pt_1_(OH)_2_Fe_2_O_3_(001) surface via C–OH bonds and Ph. Reprinted with permission [55], Copyright 2023 Elsevier. **d** XANES data of Pt/CeO_*x*_/SiO_2_. **e** EXAFS data of Pt/CeO_*x*_/SiO_2_. Reprinted with permission [62], Copyright 2025 Springer Nature. **f** COIR spectra of different samples. **g** In situ FTIR spectra revealed the conversion process of Schiff bases on the Ru_1+*n*_@Co/MMO. **h** Schematic diagram of the catalytic mechanism of the reduction amination reaction over Ru_1+n_@Co/MMO system. Reprinted with permission [46], Copyright 2024 American Chemical Society. **i** Propane conversion. **j** propylene selectivity. **k** specific activity. Reprinted with permission [56], Copyright 2023 American Chemical Society

Selective hydrogenation is a key process in fine chemical synthesis, but achieving high yields in the conversion of *α, β*-unsaturated aldehydes (UALs) to *α, β*-unsaturated alcohols (UOLs) is a major challenge due to the need for complete reduction of C = O groups while preserving the *α, β*-unsaturated C = C bonds [114, 115]. To address this issue, Liang et al. developed an innovative Pt SACs in high oxidation state by supporting functional FeO_*x*_ nanoclusters on SBA-15 (Pt_1_FeO_*x*_/SBA15) [55]. This catalyst exhibited excellent performance in the selective hydrogenation of CAL to cinnamyl alcohol (COL), with up to 95% selectivity for C = O bonds hydrogenation, while effectively inhibiting the unwanted C = C bonds reduction, and maintained 92.5% selectivity even after 48 h of continuous operation, demonstrating its excellent stability (Fig. 11a) The excellent selectivity was due to the electronic modulation of the Pt SACs through neighboring ferrite domains, as evidenced by the 1.4 e^–1^ electron transfer along the PtO_*x*_Fe interface. This electronic interaction produced different adsorption energies, with the C = O group binding stronger (Eads = −0.80 eV) than the C = C molecule (Eads = −0.68 eV) at the Pt SACs (Fig. 11b), which brought about preferential activation of C = O. Moving into Fig. 11c, the -OH at the COL terminus moved away from the catalyst surface due to the great repulsive force (Eads of −0.61 eV) between the coordinated Pt species and the -OH groups in COL. Meanwhile, the strong adsorption between the aromatic groups and the Pt SACs (Eads of 1.37 eV) resulted in that C = C had no chance to get close to the Pt SACs, which effectively inhibited their further hydrogenation. Efficient olefin hydrogenation reactions can be achieved through dynamic and stable active sites. To elucidate the structureactivity relationship of active sites, Chen et al. developed well-defined Pt/CeO_*x*_/SiO_2_ by anchoring Pt SACs to CeO_*x*_ nano-islands on SiO_2_ for the hydrogenation of ethylene [62]. The steady-state TOF characterizing Pt/CeO_*x*_/SiO_2_ at 70 °C was 0.096 ± 0.004 s^−1^. Under reaction conditions (70 °C, H_2_/ethylene/helium), the Pt species underwent dynamic reduction from initial Pt^4+^ to coordinatively unsaturated Pt^2+^ (Fig. 11d), which could serve as the active sites for reactants (H_2_ and C_2_H_4_) adsorption and activation. Crucially, the persistent absence of Pt–Pt bonding throughout the reaction confirmed the exceptional stability of isolated Pt^2+^ sites without aggregation (Fig. 11e). This unique coordination environment enabled heterolytic H_2_ dissociation, with the activated H* species simultaneously stabilizing on both Pt and adjacent oxygen sites, thereby dramatically boosting hydrogenation efficiency. Most notably, the system maintained its structural integrity even under harsh conditions in flowing H_2_, showcasing exceptional potential for industrial hydrogenation applications.

Reductive amination of aldehydes/ketones to produce primary amines represents a transformative advancement in chemical synthesis, typically involving co-adsorption of H_2_ and NH_3_ [116, 117]. The “sticking point” is the coverage imbalance caused by competitive H_2_/NH_3_ adsorption, which makes the conversion of the key intermediate Schiff bases to the more desired primary amines exponentially more difficult, replacing it with two parallel side reactions [118, 119]. The advent of SANIs has certainly significantly advanced this catalytic process. Wu et al. confined the Ru_1_ and Ru_*n*_ sites (Fig. 11f) within encapsulated Co nano-island (Ru_1+*n*_@Co/MMO) to achieve exceptional 5-aminomethyl-2-furanyl alcohol (AMF) selectivity (97%) and an impressive AMF production (295 g g_Ru_^–1^ h^–1^) [46]. The core of this exceptional performance lied in the precise site replenishment design. Specifically, the Ru_1_ sites were dedicated to H_2_ decomposition to generate H* species, while the Ru_*n*_ sites specifically catalyzed NH_3_ decomposition to generate NH_2_* intermediates. The Ru_*n*_ sites, as replenishment sites, fundamentally resolved the competitive adsorption issue between H_2_ and NH_3_, optimizing the coverage balance between H* and NH_2_*. More importantly, the confined Co nano-islands further enhanced selectivity. On one hand, it forced the Ru_1_ and Ru_*n*_ sites to be closely spaced, ensuring efficient diffusion of H* and NH_2_* species. On the other hand, the Co nano-islands provided a unique di-*σ*C,N adsorption configuration (Fig. 11g), offering specific adsorption sites for Schiff base intermediates, ensuring smooth progression of subsequent ammonia decomposition and hydrogen decomposition steps, and efficiently guiding the formation of primary amine products (Fig. 11h).

More recently, SANIs have shown tremendous promise in propane dehydrogenation. Wang and co-workers developed SiO_2_-supported InCeO_*x*_ nano-island system that confined isolated Pt^δ+^ sites (denoted as 0.5Pt0.3In(2.0Ce)/SiO_2_) to break the constraints of the activity-selectivity-stability-constraints in the dehydrogenation of propane [56]. The performance of 0.5Pt0.3In(2.0Ce)/SiO_2_ was excellent, with propane conversion of 67.1%, propylene selectivity of 92.2%, and deactivation constants as low as 0.010 h^–1^ (Fig. 11i, j). The enhanced stability and activity stemmed from the strategic integration of InCeO_*x*_ nano-islands inhibiting the agglomeration and carbon deposition of Pt^δ+^. In addition, the addition of Ce modulated the electronic interactions between the Pt and InCeO_*x*_ substrates, thereby optimizing the adsorptiondesorption behavior of propane on the 0.5Pt0.3In(2.0Ce)/SiO_2_ surface. This electronic tailoring reduced the carbon depositions caused by the excessive adsorption of intermediates, thus effectively inhibiting coking.

### Carbon Dioxide Reduction Reaction

**Fig. 12:**
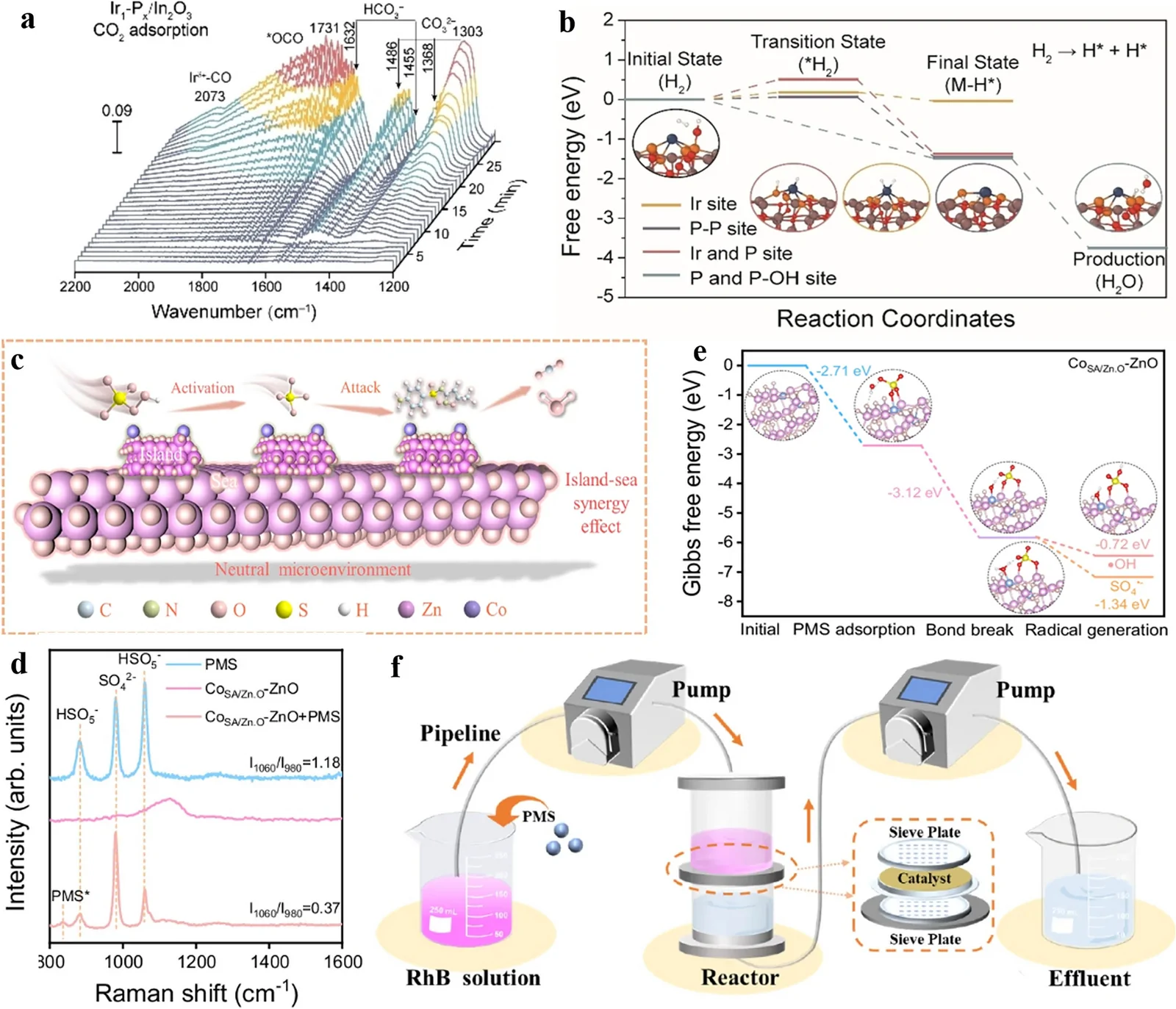
Employing SANIs in other catalysis. **a** In situ DRIFTS spectra over Ir_1_-P_x_/In_2_O_3_ exposed to CO_2_ for 30 min at 180 °C. **b** Energy diagram for the process H_2_ → H* + H* occurring at Ir/P sites (red), P/P-OH sites (blue), P/P sites (purple), and Ir sites (yellow) on Ir_1_-P_*x*_/In_2_O_3_. Reprinted with permission [57], Copyright 2025 Wiley. **c** Reaction mechanisms in the Co_SA/Zn.O_-ZnO/PMS system. **d** In situ Raman spectra of different reaction systems. **e** Gibbs free energy of PMS activation Co_SA/Zn.O_-ZnO. **f** Schematic diagram of continuous flow reactor. Reprinted with permission [35], Copyright 2025 Springer Nature

In the face of the urgent challenge of combating global warming, the carbon dioxide reduction reaction (CO_2_RR), as a cutting-edge technology, is gradually unveiling the green revolution of turning greenhouse gases into valuable resources. In a novel Ir_1_/P*ₓ*/In_2_O_3_ catalyst [57], phosphorus cluster islands (P islands) integrated with IrSACs on In_2_O_3_ nanosheets achieved an ethanol yield of 3.33 mmol g^–1^ h^–1^ and a TOF of 914 h^–1^. Ir_1_/P*ₓ*/In_2_O_3_ exhibited nearly 8 times higher performance compared to unmodified Ir_1_/P*ₓ*/In_2_O_3_, attributed to the dual role of P islands in the constructed system: (i) electronic regulation. Ir SACs served as the primary active sites in the systems, responsible for CO_2_ adsorption, activation, and C–C coupling processes (Fig. 12a). In RLS (*OCHO → *OCH_2_O, P islands modulated the electronic structure of Ir SACs through specific coordination, significantly reducing the energy barrier and thereby significantly promoting the reaction process; and (ii) sites complementation. P islands were highly efficient hydrogen activators, with their P/POH sites exhibiting the lowest hydrogen activation energy barriers (Fig. 12b). These complementation sites compensated for the limited hydrogenation capacity of Ir SACs by supplying H* to adsorbed CO_2_ intermediates. Ir SACs and P islands exhibited clear division of labor and perfect synergy, achieving efficient CO_2_RR performance.

### Advanced Oxidation Processes

PMS-AOPs, with its characteristics of high efficiency, flexibility, and environmental friendliness, demonstrates significant advantages in the removal of refractory pollutants, offering innovative solutions for water pollution control and ecological restoration. Recent breakthroughs in nano-island architecture design have revolutionized heterogeneous catalyst development for PMSAOPs. Yu et al. creatively loaded Co SACs onto islands with ZnO NPs acting as sea and island (Co_SA/Zn.O_ZnO), achieving superior catalytic efficiency and remarkable operational stability, which successfully solved the activity-stability challenge in SACs (Fig. 12c) [35]. The excellent performance stemmed from structural modifications at the atomic level, i.e., Co substitution at the Zn site induced an asymmetric charge redistribution, which triggered a significant spin polarization and an elevated position of the center of the Co d-band. These electronic modifications enhanced the adsorption capacity of PMS and promoted the efficient cleavage of O–O bonds (Fig. 12d), while preferentially generating highly-oxidative SO_4_^•−^ (Fig. 12e). This superb stability was attributed to the fact that ZnO nano-islands could firmly anchor dispersed Co SACs, which resisted detachment during PMS activation. Meanwhile, the ZnO supports (“sea”) regulated the reaction pH and maintained a neutral microenvironment. Notably, the system demonstrated substantial industrialization potential, maintaining a high degradation rate of 95% for SMX and complete degradation of rhodamine B (RhB) within 12 h using a continuousflow reactor (Fig. 12f). Concurrently, it exhibited significant treatment efficacy for real coking wastewater. These achievements marked a substantial breakthrough for the catalytic system in the fields of long-term pollutant degradation and wastewater treatment applications.

## Concluding Remarks and Perspectives

This study provides a comprehensive exploration of SANIs as innovative catalytic structures, focusing on their precise synthesis, “moving but not aggregation” dynamic behavior, inter-system interactions, and their transformative potential for multidisciplinary applications. By combining atomic-scale dispersion with nano-island-mediated stabilization, SANIs go beyond the classical activity-stability trade-off of conventional SACs and provide a versatile and efficient catalytic platform for various catalytic fields. The effect of stabilization and sintering on the sites was overcome. At the same time, the nano-islands optimize the physicochemical, geometrical and electronic structures of the SACs sites to achieve dynamic adaptability under harsh conditions, showing excellent performance. Notably, the synergy between atomic precision and nanoscale confinement expands the reaction versatility and selectivity, unleashing unprecedented catalytic versatility.

**Fig. 13:**
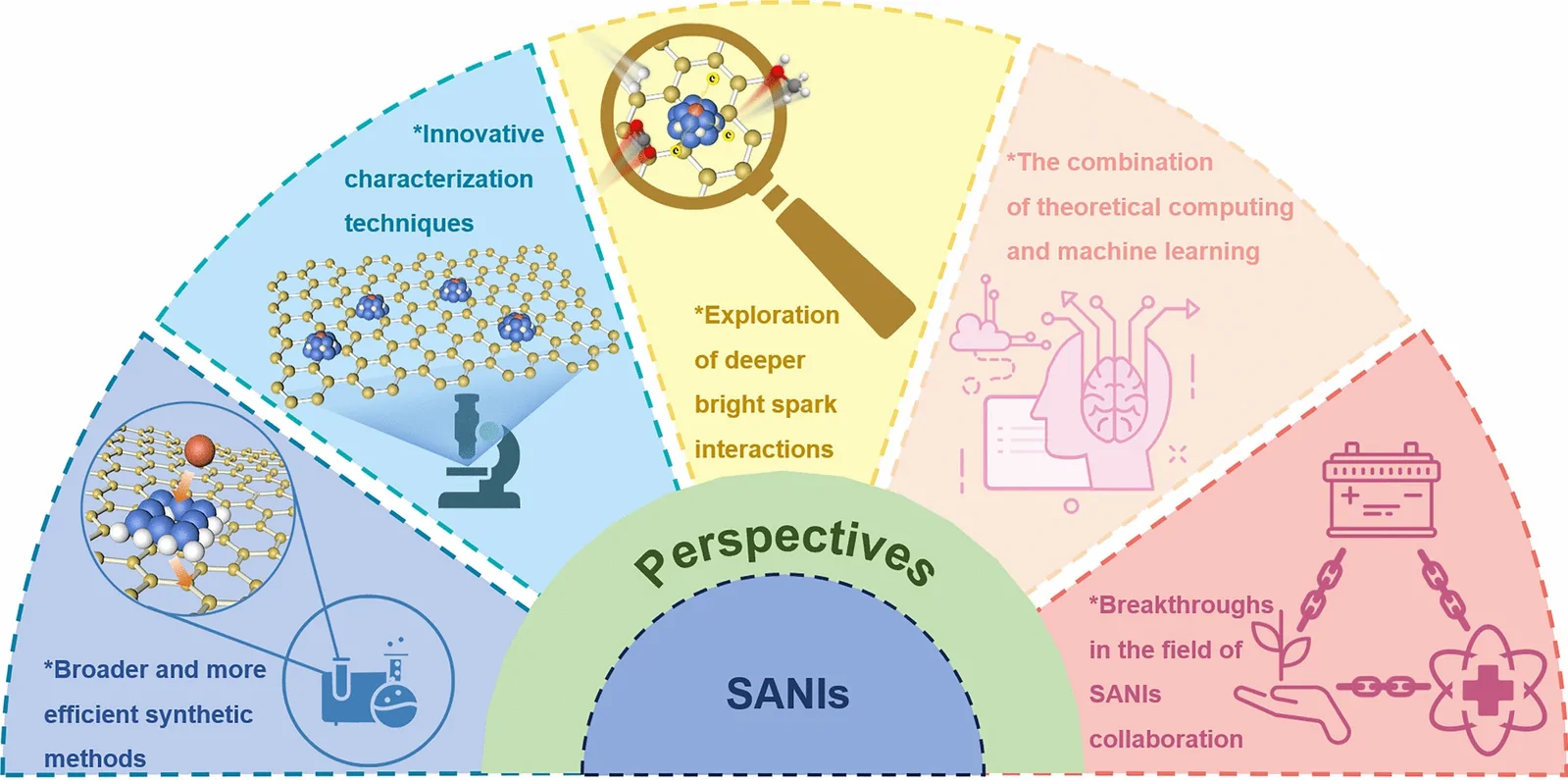
Summary of the perspectives for SANIs

Although great progress has been made in the use of SANIS as catalysts, the following challenges still need to be addressed in this field for future research and interdisciplinary (Fig. 13).

### Broader and More Efficient Synthetic Methods for SANIs

The development of broader and more efficient synthesis strategies for SANIs is conducive to their broader applications. Electric pulse and microwave-assisted methods address the limitations of pyrolysis methods [120–122], but also face stringent support selection. Future work should focus on hybrid support design, such as constructing conductive-polar hybrid composite supports or designing defect-rich substrates to improve compatibility. Notably, emerging photochemical reduction and strong electrostatic adsorption technologies offer new insights for the precise construction of SANIs. For example, the semiconductor nano-islands can enable light-induced SAs deposition, while regulating the isoelectric point difference between nano-islands and the supports can achieve selective adsorption and localization of metalprecursors. Meanwhile, atomic layer deposition (ALD) and chemical vapor deposition (CVD) should be further explored to optimize the uniform nucleation and growth of SACs on nano-islands. It is also critical to advance scalable and energy-efficient synthesis processes. This includes designing continuousflow electric pulse or microwave reaction systems and improving energy parameters (e.g., pulse duration and microwave power) to minimize energy consumption, thereby addressing the cost gap issue when scaling up from laboratory to industrial applications. It is particularly important to note that during scaling, challenges related to maintaining active sites stability, supports durability, and selectivity must be addressed simultaneously to ensure that the performance of industrial-scale SANIs electrocatalysts does not degrade. Another key challenge lies in the issue of experimental reproducibility, that is, inconsistent results may emerge in material characterization and performance evaluation across different studies, which could constrain the large-scale application of SANIs. Therefore, it is necessary to establish a standardized synthesis protocol for SANIs, clearly defining critical parameters and their tolerance ranges. Additionally, it is essential to develop a modular synthesis platform and promote the use of commercial substrate materials (e.g., standard carbon paper and metal foils) to reduce the variability introduced by substrate pretreatment.

### Innovative Characterization Techniques Applied to SANIs

Advances in characterization techniques have been crucial in unraveling the structural and functional complexity of SANIs. Although existing techniques have partially elucidated their properties, they still face significant limitations. The signal ambiguity at the interface between SACs and nano-islands often leads to false-positive results, and static characterization struggles to capture the dynamic evolution of active sites. Especially for electron-beam-sensitive materials, high-dose or prolonged exposure in situ TEM characterization may cause structural damage and distort the true structure–property relationship. The limitations of characterization techniques may also lead to a limited resolution in analyzing the local coordination environment, resulting in a distorted understanding of the structure of SANIs and thereby further undermining experimental reproducibility. Future research must priorities the development of atomically precise, low-disturbance methods, such as using aberration-corrected HAADF-STEM combined with artificial intelligence (AI) image analysis to reliably distinguish active sites. Concurrently, low-dose, time-resolved TEM techniques should be developed to resolve the dynamic evolution of active sites while minimizing irradiation damage. Integration of operational spectroscopy (e.g., in situ XAS, Raman) and time-resolved microscopy will elucidate real-time structural dynamics and reaction mechanisms. These innovations will bridge the gap between synthetic design and performance optimization in complex catalytic systems.

### Exploration of Deeper Bright Spark Interactions in SANIs

Despite the progress made in the catalytic mechanism of SANIs, key uncertainties remain. A key challenge lies in deciphering how the atomic-level coordination environments (e.g., bond lengths and lattice strain) and electronic states (e.g., charge transfer and d-band centers) at the nano-island/SACs interfaces dynamically evolve under operating conditions. In addition, synergistic interactions between nano-islands and SACs, such as electron redistribution through interfacial “spillover” effects or strain-induced activation, remain under-explored. The size effect of nano-island supports is also worthy of investigation regarding the modulation of SA electronic states. Variations in the support size can adjust the electron interaction and spin state between SAs and nano-islands, altering the occupation states and chemical properties of SAs. In the future, it is essential to integrate experimental characterization with theoretical calculations to establish a quantitative relationship model between nano-island size and SACs electronic states. Additionally, apart from nano-island size, the rational design of their crystallinity and defects can modulate the MSIs, thereby enhancing the cyclic stability of SANIs. It is important to note that elucidating the unique synergistic mechanisms of different SANIs models is a key pathway to breaking through performance boundaries, as their configurational effects directly determine the scale of electron transfer and atomic motion freedom. Therefore, future research should also focus more on the potential mechanisms underlying the synergistic catalytic activity of SACs and nano-islands in different types of SANIs. In particular, in situ or operational condition experiments should be conducted to uncover the regulatory patterns of nano-island structural features (such as size, crystallinity, and defects) on SACs reactivity, thereby guiding the rational design of layered structures, enhancing MSIs, and ultimately achieving a comprehensive improvement in catalytic activity, selectivity, and stability. Notably, recent studies have confirmed that the synergistic interaction between atomic clusters and nano-islands can significantly enhance catalytic performance. Future research can extend SANIs to atomic clusters and nano-islands to promote broader applications in this field.

### The Combination of Theoretical Computing and Machine Learning (ML) Drives SANIs

Although DFT plays an important role in deciphering atomic-scale interactions and guiding catalyst design. However, its limitations (e.g., parameter sensitivity, high computational cost, and static approximations) prevent accurate modeling of the dynamic behavior of SANIs under realistic conditions. To address these challenges, future research should prioritize multi-scale computational frameworks that synergize DFT with ML-driven surrogate models. For example, ML algorithms trained on high-throughput datasets (e.g., nano-island sizes, defect densities, and metalligand environments) and dynamic Monte Carlo simulation can rapidly predict optimal SANIs configurations, while graph neural networks (GNNs) can decode complex structure–activity relationships across hierarchical interfaces, thereby breaking through the ultrastable design of supported nano-catalysts [123]. In addition, embedded operational simulations (e.g., atomic molecular dynamics under electric/thermal field conditions) and interpretable artificial intelligence (AI) tools will bridge the gap between static predictions and dynamic catalytic reality. Importantly, it is also necessary to promptly carry out detailed experimental characterizations and performance tests to further validate the prediction results of ML models and continuously optimize the models based on experimental feedback. The closed-loop research system of “ML prediction-high-throughput screening-experimental validation-model optimization” will accelerate the rational design of SANIs with customized nano-island architectures and atomic-level precision, ultimately enabling unprecedented catalytic efficiency and stability.

### Breakthroughs in the Field of Sanis Collaboration

The future development of SANIs must fully leverage their atomic-level precision, tunable coordination environments, and interfacial synergies to expand application scope from traditional thermal-/photo-/electrocatalysis to emerging directions such as piezoelectric catalysis and plasma-assisted catalysis. Innovations should target interdisciplinary frontiers such as quantum technology and biomedicine, where isolated atomic spins on nano-island enable scalable arrays of quantum bits. In biomedicine, enzyme-mimicking nano-island can drive targeted therapies or ultra-sensitive biosensing. In energy storage, SACs enclosed in nano-islands could revolutionize high-capacity battery electrodes or oxygen catalysis. Achieving these goals requires interdisciplinary convergence, integrating atomic-scale materials design, AI-accelerated simulation, and modular reactor engineering to address core challenges in scalable fabrication, operational stability, and multifunctional integration. By coordinating these advances, SANIs will transition from a lab-scale curiosity to an industrial pillar in clean energy, smart electronics, and more, ultimately bridging the gap between atomic innovation and the needs of global sustainability.
